# NaV1.5 autoantibodies in Brugada syndrome: pathogenetic implications

**DOI:** 10.1093/eurheartj/ehae480

**Published:** 2024-07-30

**Authors:** Adriana Tarantino, Giuseppe Ciconte, Dario Melgari, Anthony Frosio, Andrea Ghiroldi, Marco Piccoli, Marco Villa, Pasquale Creo, Serena Calamaio, Valerio Castoldi, Simona Coviello, Emanuele Micaglio, Federica Cirillo, Emanuela Teresina Locati, Gabriele Negro, Antonio Boccellino, Flavio Mastrocinque, Žarko Ćalović, Stefano Ricagno, Letizia Leocani, Gabriele Vicedomini, Vincenzo Santinelli, Ilaria Rivolta, Luigi Anastasia, Carlo Pappone

**Affiliations:** Institute for Molecular and Translational Cardiology (IMTC), IRCCS Policlinico San Donato, Piazza Malan, 2, 20097 San Donato Milanese, Milan, Italy; School of Medicine, University Vita-Salute San Raffaele, Via Olgettina, 58, 20132 Milan, Italy; Institute for Molecular and Translational Cardiology (IMTC), IRCCS Policlinico San Donato, Piazza Malan, 2, 20097 San Donato Milanese, Milan, Italy; School of Medicine, University Vita-Salute San Raffaele, Via Olgettina, 58, 20132 Milan, Italy; Arrhythmology Department, IRCCS Policlinico San Donato, Piazza Malan, 2, 20097 San Donato Milanese, Milan, Italy; Institute for Molecular and Translational Cardiology (IMTC), IRCCS Policlinico San Donato, Piazza Malan, 2, 20097 San Donato Milanese, Milan, Italy; Institute for Molecular and Translational Cardiology (IMTC), IRCCS Policlinico San Donato, Piazza Malan, 2, 20097 San Donato Milanese, Milan, Italy; Institute for Molecular and Translational Cardiology (IMTC), IRCCS Policlinico San Donato, Piazza Malan, 2, 20097 San Donato Milanese, Milan, Italy; Institute for Molecular and Translational Cardiology (IMTC), IRCCS Policlinico San Donato, Piazza Malan, 2, 20097 San Donato Milanese, Milan, Italy; Institute for Molecular and Translational Cardiology (IMTC), IRCCS Policlinico San Donato, Piazza Malan, 2, 20097 San Donato Milanese, Milan, Italy; Institute for Molecular and Translational Cardiology (IMTC), IRCCS Policlinico San Donato, Piazza Malan, 2, 20097 San Donato Milanese, Milan, Italy; Institute for Molecular and Translational Cardiology (IMTC), IRCCS Policlinico San Donato, Piazza Malan, 2, 20097 San Donato Milanese, Milan, Italy; Experimental Neurophysiology Unit, Institute of Experimental Neurology-INSPE, IRCCS Ospedale San Raffaele, Via Olgettina, 58, 20132 Milan, Italy; Institute for Molecular and Translational Cardiology (IMTC), IRCCS Policlinico San Donato, Piazza Malan, 2, 20097 San Donato Milanese, Milan, Italy; Institute for Molecular and Translational Cardiology (IMTC), IRCCS Policlinico San Donato, Piazza Malan, 2, 20097 San Donato Milanese, Milan, Italy; Arrhythmology Department, IRCCS Policlinico San Donato, Piazza Malan, 2, 20097 San Donato Milanese, Milan, Italy; Institute for Molecular and Translational Cardiology (IMTC), IRCCS Policlinico San Donato, Piazza Malan, 2, 20097 San Donato Milanese, Milan, Italy; Institute for Molecular and Translational Cardiology (IMTC), IRCCS Policlinico San Donato, Piazza Malan, 2, 20097 San Donato Milanese, Milan, Italy; Arrhythmology Department, IRCCS Policlinico San Donato, Piazza Malan, 2, 20097 San Donato Milanese, Milan, Italy; Institute for Molecular and Translational Cardiology (IMTC), IRCCS Policlinico San Donato, Piazza Malan, 2, 20097 San Donato Milanese, Milan, Italy; Arrhythmology Department, IRCCS Policlinico San Donato, Piazza Malan, 2, 20097 San Donato Milanese, Milan, Italy; Institute for Molecular and Translational Cardiology (IMTC), IRCCS Policlinico San Donato, Piazza Malan, 2, 20097 San Donato Milanese, Milan, Italy; Arrhythmology Department, IRCCS Policlinico San Donato, Piazza Malan, 2, 20097 San Donato Milanese, Milan, Italy; Institute for Molecular and Translational Cardiology (IMTC), IRCCS Policlinico San Donato, Piazza Malan, 2, 20097 San Donato Milanese, Milan, Italy; Arrhythmology Department, IRCCS Policlinico San Donato, Piazza Malan, 2, 20097 San Donato Milanese, Milan, Italy; Arrhythmology Department, IRCCS Policlinico San Donato, Piazza Malan, 2, 20097 San Donato Milanese, Milan, Italy; Institute for Molecular and Translational Cardiology (IMTC), IRCCS Policlinico San Donato, Piazza Malan, 2, 20097 San Donato Milanese, Milan, Italy; Department of Biosciences, Università degli Studi di Milano, 20133 Milan, Italy; School of Medicine, University Vita-Salute San Raffaele, Via Olgettina, 58, 20132 Milan, Italy; Experimental Neurophysiology Unit, Institute of Experimental Neurology-INSPE, IRCCS Ospedale San Raffaele, Via Olgettina, 58, 20132 Milan, Italy; Institute for Molecular and Translational Cardiology (IMTC), IRCCS Policlinico San Donato, Piazza Malan, 2, 20097 San Donato Milanese, Milan, Italy; Arrhythmology Department, IRCCS Policlinico San Donato, Piazza Malan, 2, 20097 San Donato Milanese, Milan, Italy; Arrhythmology Department, IRCCS Policlinico San Donato, Piazza Malan, 2, 20097 San Donato Milanese, Milan, Italy; Institute for Molecular and Translational Cardiology (IMTC), IRCCS Policlinico San Donato, Piazza Malan, 2, 20097 San Donato Milanese, Milan, Italy; School of Medicine and Surgery, University of Milano-Bicocca, Via Cadore, 48, 20900 Monza, Italy; Institute for Molecular and Translational Cardiology (IMTC), IRCCS Policlinico San Donato, Piazza Malan, 2, 20097 San Donato Milanese, Milan, Italy; School of Medicine, University Vita-Salute San Raffaele, Via Olgettina, 58, 20132 Milan, Italy; Institute for Molecular and Translational Cardiology (IMTC), IRCCS Policlinico San Donato, Piazza Malan, 2, 20097 San Donato Milanese, Milan, Italy; School of Medicine, University Vita-Salute San Raffaele, Via Olgettina, 58, 20132 Milan, Italy; Arrhythmology Department, IRCCS Policlinico San Donato, Piazza Malan, 2, 20097 San Donato Milanese, Milan, Italy

**Keywords:** Brugada syndrome, Autoantibodies, NaV1.5, Biomarker

## Abstract

**Background and Aims:**

Patients suffering from Brugada syndrome (BrS) are predisposed to life-threatening cardiac arrhythmias. Diagnosis is challenging due to the elusive electrocardiographic (ECG) signature that often requires unconventional ECG lead placement and drug challenges to be detected. Although NaV1.5 sodium channel dysfunction is a recognized pathophysiological mechanism in BrS, only 25% of patients have detectable *SCN5A* variants. Given the emerging role of autoimmunity in cardiac ion channel function, this study explores the presence and potential impact of anti-NaV1.5 autoantibodies in BrS patients.

**Methods:**

Using engineered HEK293A cells expressing recombinant NaV1.5 protein, plasma from 50 BrS patients and 50 controls was screened for anti-NaV1.5 autoantibodies via western blot, with specificity confirmed by immunoprecipitation and immunofluorescence. The impact of these autoantibodies on sodium current density and their pathophysiological effects were assessed in cellular models and through plasma injection in wild-type mice.

**Results:**

Anti-NaV1.5 autoantibodies were detected in 90% of BrS patients vs. 6% of controls, yielding a diagnostic area under the curve of .92, with 94% specificity and 90% sensitivity. These findings were consistent across varying patient demographics and independent of *SCN5A* mutation status. Electrophysiological studies demonstrated a significant reduction specifically in sodium current density. Notably, mice injected with BrS plasma showed Brugada-like ECG abnormalities, supporting the pathogenic role of these autoantibodies.

**Conclusions:**

The study demonstrates the presence of anti-NaV1.5 autoantibodies in the majority of BrS patients, suggesting an immunopathogenic component of the syndrome beyond genetic predispositions. These autoantibodies, which could serve as additional diagnostic markers, also prompt reconsideration of the underlying mechanisms of BrS, as evidenced by their role in inducing the ECG signature of the syndrome in wild-type mice. These findings encourage a more comprehensive diagnostic approach and point to new avenues for therapeutic research.

## Structured Graphical Abstract

**Structured Graphical Abstract:**
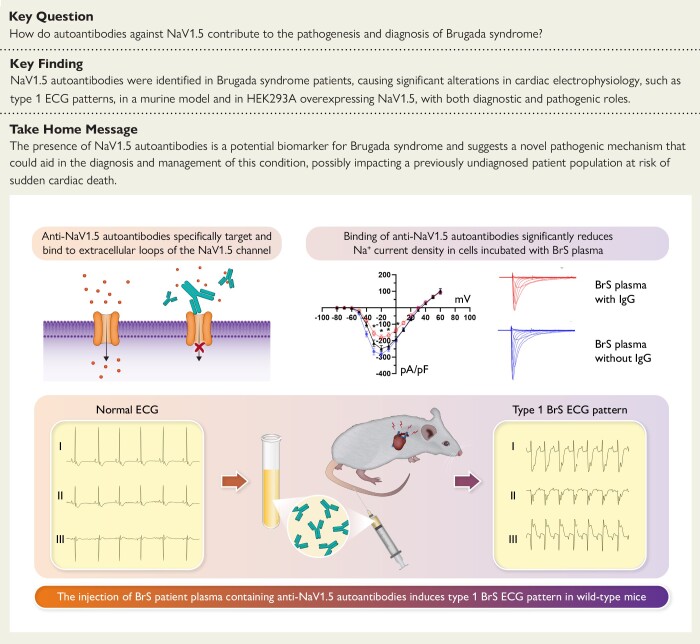
Role of anti-NaV1.5 autoantibodies in Brugada syndrome. It demonstrates how these autoantibodies, when present in patient plasma, can elicit the coved-type ST-segment elevation resembling the human type 1 Brugada syndrome electrocardiographic pattern in wild-type mice and significantly reduce sodium current density, supporting their diagnostic and pathogenic significance in the disease. BrS, Brugada syndrome; ECG, electrocardiographic; IgG, immunoglobulin G.

**See the editorial comment for this article ‘Cardiac arrhythmias: the growing role of autoantibodies in diagnosis and treatment’, by F.E. Fakuade *et al*., https://doi.org/10.1093/eurheartj/ehae648.**

**Translational perspective**

In a significant shift from the translational perspective to Brugada syndrome (BrS), this study highlights the role of autoimmunity by identifying anti-NaV1.5 autoantibodies in affected patients, including those without *SCN5A* mutations. This discovery is set to complement previous diagnostics based on electrocardiographic manifestations and drug testing and provides a reliable, non-invasive biomarker but also calls for a re-evaluation of the pathophysiology of BrS involving immune-mediated mechanisms. The potential of immunomodulatory therapies, especially for genetically elusive cases, may introduce a new era of personalized treatment strategies.

## Introduction

Brugada syndrome (BrS) is an inherited arrhythmic cardiomyopathy that can lead to ventricular arrhythmia and sudden cardiac death.^1^ Its diagnostic hallmark is an electrocardiogram (ECG) displaying a distinct ST-segment elevation known as the type 1 BrS pattern.^2^ However, nearly half of BrS patients surviving cardiac arrest exhibit normal ECGs, with only transient expression of the ECG pattern.^3,4^ While the type 1 pattern can sometimes be revealed using sodium channel blockers (SCBs),^5^ this approach has significant limitations.^6^ The pro-arrhythmic potential of such drugs, which are often not available in various countries, and the need for special cardiac monitoring for their administration limit their widespread use.^7^ These compounding challenges are ongoing concerns regarding the true specificity and sensitivity of these drug tests.^6^ Therefore, the difficulties in consistently detecting the diagnostic ECG pattern associated with the genetic inheritance of BrS^2,8,9^ point to an inadequate estimate of the true prevalence of the disease. While more than two dozen genes are associated with BrS, the *SCN5A* gene, which encodes the alpha subunit of the cardiac voltage-gated sodium channel NaV1.5, takes centre stage.^9–13^ About 20%–25% of BrS diagnoses are associated with variants in this gene, but the genetic basis for the remaining majority, almost 70%–75%, remains unknown.^14,15^ In addition to *SCN5A*, other genes, including those related to sodium channel β-subunits and potassium and calcium channel genes, have also been investigated for their possible involvement, suggesting a broad and complex genetic basis for the syndrome.^14,15^ Nevertheless, the clinical relevance of variations in these additional genes is frequently debated, highlighting the challenges that genetic testing faces in definitively diagnosing a significant proportion of BrS cases.^15,16^ To address the complexities associated with genotype–phenotype correlation in BrS, a comprehensive scoring system was developed to aid clinicians identify BrS patients.^2,17^ Nevertheless, the integration of ECG recordings, genetic information, clinical characteristics, and family history into the diagnostic process for BrS is intricate. This complexity underscores the need for an enhanced understanding of the molecular basis of the disease. Along this line, histological analyses of *ex vivo* heart tissues from BrS patients have revealed abnormalities such as inflammation, fibrosis, and fatty infiltration in the epicardial region of the right ventricle, alongside the detection of autoantibodies against some common cardiac proteins.^18–23^ These observations resonate with the growing recognition of immune system involvement in various cardiac disorders, including heart failure,^24^ long QT,^25^ myocarditis,^26^ arrhythmogenic right ventricular cardiomyopathy,^27^ atrioventricular (AV) block,^28^ and atrial fibrillation.^29^ Despite increasing recognition of the immune system’s role in cardiac disorders,^30^ the exact contribution to the pathogenesis of BrS remains poorly defined,^23^ as it is considered a channelopathy associated with mutations in the NaV1.5 channel.

Given the possibility that an autoimmune component contributes to the complexity of the disease and the challenges in identifying a consistent genetic profile for BrS, we noted that there is no evidence in the literature of NaV1.5 autoantibodies in BrS patients. Therefore, our study was designed to investigate the presence of anti-NaV1.5 autoantibodies in BrS and determine whether they contribute to the pathogenesis of the disease.

## Methods

### Human samples

Subjects included in the study were all referred to the Arrhythmology Department of the IRCCS Policlinico San Donato for investigation of BrS. Patients in the BrS group were diagnosed according to current consensus statements,^2,31^ while the control group was composed of subjects with a negative BrS diagnosis, as confirmed by the SCB test.^2,31^ Additional control plasma were collected from a cohort of 35 patients admitted to our institution who were diagnosed with other cardiac conditions, such as long QT syndrome, structural cardiomyopathies, and heart failure. Further details on the diagnostic criteria for BrS and the inclusion and exclusion criteria of the two study groups can be found in the Supplementary data. The protocol of this study was reviewed and approved by the local Institutional Ethics Committee, and all participants provided written informed consent, in compliance with the Declaration of Helsinki. All authors had full access to all data in the study and take responsibility for its integrity and data analysis.

### Plasma collection

Blood samples (25 mL) were centrifuged at 1000 × *g* for 15 min to separate plasma. A second centrifugation at 2000 × *g* for 15 min further clarified the plasma, which was then aliquoted and stored at −20°C.

### Immunoglobulin G plasma depletion

Two hundred microliters of plasma from BrS patients were incubated with Protein G magnetic Beads (Thermo Fisher) for 1.5 h at room temperature in a rotator. The procedure was repeated three times with fresh beads to ensure removal of the immunoglobulin G (IgG) fraction.

### Generation of stable cell line and transient transfections

HEK293A cells were cultured in Dulbecco’s Modified Eagle Medium (DMEM, Life Technologies) supplemented with 10% foetal bovine serum (FBS, Sigma), 2 mM glutamine (Merck), and 1× penicillin/streptomycin (Euroclone), at 37°C in a humidified atmosphere of 5% CO_2_ and 95% air. The NaV1.5 plasmid was synthesized, encoding the full-length human NaV1.5 cDNA, and cloned into the pcDNA 3.1(+) vector. Transfections were performed using jetPRIME (Euroclone), following the manufacturer’s protocol, with cells later selected using G418. HEK293T stable over-expressing CaV3.2 calcium channel was cultured in DMEM high glucose as described above, the pharma-selection was done in 100 μg/μL hygromycin B and 15 μg/µL blasticidin S hydrochloride, and protein CaV3.2 induction was done 24 h before the day of the patch clamp experiments using tetracycline 2.5 μg/μL.

### NaV1.5 immunoblotting

HEK293A cells over-expressing NaV1.5 were lysed using RIPA buffer containing a cocktail of protease and phosphatase inhibitors, with subsequent centrifugation at 15 000 rpm for 10 min at 4°C to collect the supernatant. Protein concentrations were determined by the bicinchoninic acid assay (Pierce). Proteins were denatured and reduced with Laemmli buffer containing β-mercaptoethanol (Bio-Rad) and loaded onto a 10% SDS-PAGE gel (Protean Tgx Stain-Free, Bio-Rad) for electrophoresis, followed by transfer to nitrocellulose membranes. The membranes were blocked and probed overnight at 4°C with the primary anti-NaV1.5 antibody (1:2000 dilution, Cell Signaling, clone D9J7S), followed by washing and incubation with a secondary anti-rabbit IRDye 800 CW antibody (1:2000 dilution, LI-COR Biosciences). Membranes were then incubated with BrS patient or control plasma, followed by staining with a secondary anti-human IgG-HRP antibody (1:800 dilution, Bio-Rad). Bands were visualized using the ECL Advance Kit (GE Healthcare) and imaged with the ChemiDoc MP system (Bio-Rad). Experiments were conducted in a double-blind manner by five independent researchers (A.T., A.G., S.C., M.P., and F.C.).

### Cell-based immunofluorescence assay

Plasma positive for IgGs-NaV1.5 from BrS patients was tested on live MOCK-HEK293A, and HEK293A cells over-expressing NaV1.5. 2 × 10^5^ cells were plated on a coverslip the day before the experiment, and after 24 h, cells were incubated with plasma from BrS patients and controls for 1 h. After two washes with phosphate-buffered saline (PBS) 1×, cells were fixed in 4% paraformaldehyde for 15 min at room temperature. Subsequently, cells were incubated in a blocking solution [3% bovine serum albumin (BSA) and 2% normal donkey serum (NDS)] for 1 h and stained with NaV1.5 antibody (Cell Signaling) diluted at 1:500 for 2 h. After multiple washes, secondary antibodies, including anti-rabbit Alexa Fluor 594 (Thermo Fisher) diluted at 1:500 and anti-human IgG-FITC (Bio-Rad) diluted at 1:500, were added for 1 h at room temperature. Nucleus staining was performed with DAPI staining solution for 5 min at room temperature, followed by several washes before image capture using the Leica Thunder Imager System (×40 objective). Co-localization was assessed using the JACoP plugin on FIJI software.^32^

### Cellular electrophysiology

NaV1.5-GFP plasmid^33^ was kindly supplied by Sebastien Roger from the University of Tours, France.

The chip-based automated planar patch-clamp system Patchliner (Nanion Technologies GmbH, Munchen, Germany) was employed to record sodium currents from HEK293A cells transiently transfected with the NaV1.5 channel. After 1-h incubation at 37°C and 5% CO_2_ with 5% BrS- or control-derived plasma (see Supplementary data), cells were gently tripsinized (1 min at 37°C with .05% Trypsin-EDTA, by Gibco code 25300-054) and re-suspended in the extracellular low-sodium recording solution from Nanion (Ref. 08-3004, ionic composition in mM: 80 NaCl, 60 NMDG, 4 KCl, 2 CaCl_2_, 1 MgCl_2_, 5 D-glucose monohydrate, 10 Hepes; pH 7.4 with HCl, 289 mOsm). Untreated cells were used as internal reference. To improve membrane stability, HEK293A cells over-expressing NaV1.5 protein were then incubated at 4°C for 20 min. All recordings were performed at room temperature in whole-cell configuration using medium resistance NPC-16 chips. At least two chips for the condition were used on each experimental day. CsF-based intracellular (Ref. 08-3008, ionic composition in mM: 10 EGTA, 10 Hepes, 10 CsCl, 10 NaCl, 110 CsF; pH 7.2 with CsOH, 280 mOsm) and the extracellular seal enhancer (Ref. 08-3011, ionic composition in mM: 80 NaCl, 60 NMDG, 4 KCl, 10 CaCl_2_, 1 MgCl_2_, 5 D-glucose monohydrate, 10 Hepes; pH 7.4 with HCl, 313 mOsm) solutions were also provided from Nanion. To reduce any bias due to transfection variability, at least one BrS plasma and one control plasma from healthy donors were tested on each individual experimental day. To confirm that the observed effect on NaV1.5 current was related to IgG, total IgGs were removed from the plasma of three BrS patients (see Methods). Cell capacitance and series resistance were automatically compensated by the Patchliner at 80%–90%. All currents were sampled at 50 kHz. The current–voltage relationship and the voltage dependence of activation of the sodium currents were obtained applying a protocol with incremental 50-ms steps ranging from −80 to +60 mV (holding potential −120 mV). The steady-state inactivation protocol had an initial 500 ms duration step (from −140 mV to +10 mV, increments of +10 mV), followed by a −10 mV test pulse (20-ms durations). Raw traces recorded by HEK293A amplifiers were exported with a home-built Python tool, and individual traces were analysed using Clampfit 10.7 (Molecular Devices, San Jose, CA, USA), Origin Pro (OriginLab, Northampton, MA, USA), and GraphPad Prism (GraphPad Software, Boston, MA, USA). Current density was calculated dividing the current amplitude (pA) by the cell capacitance (pF) for each cell. Steady-state activation and inactivation curves were fitted with a Boltzmann function: *y* = 1/(1 + exp((*V* − *V*_1/2_)/*k*)), where *y* is the relative current, *V* is the membrane potential, *V*_1/2_ is the half-maximal voltage, and *k* is the slope factor. All electrophysiological experiments were performed without prior knowledge of the origin of the plasma samples to ensure the integrity of the results.

### Animals and electrocardiography

The procedure involving mice was performed according to the animal protocol guidelines described by the Institutional Animal Care and Use Committee authorization no. 425/2022/PR at San Raffaele Scientific Institute (Milan, Italy). Mice C57BL-6 at 50 weeks were maintained *ad libitum* access to water and standard chow food at room temperature with a 12-h light/dark schedule. They were anesthetized by intra-peritoneal injection of medetomidine, .5 mg/kg (Orion Pharma s.r.l.) and ketamine, 100 mg/kg (Merial), both diluted in saline solution. Body weight was determined prior to each investigation; the body temperature was constantly monitored and kept at 37 ± .5°C by a homoeothermic blanket system with a rectal thermometer probe (Harvard Apparatus, Holliston, MA, USA). Two hundred microliters of plasma from BrS (*n* = 4) and control (*n* = 3) were pre-heated at 56°C for 20 min before the intravenous injection in anesthetized mice. Briefly, ECG was performed continuously from 10 min after induction of anaesthesia and 30 min after plasma injection using four subcutaneous needle electrodes (stainless steel, 27-G, 12 mm length; SEI EMG s.r.l., Cittadella, Italy): two needles were inserted in the forelimbs and one in the left hindlimb, and another needle electrode was placed in the right hindlimb as a ground. A consistent lead configuration was used without changing the polarity or placement of the subcutaneous needle electrodes before, during, and after plasma administration. Specifically, the leads were placed in the standard Einthoven configuration for mice, ensuring that each mouse underwent the same procedure for accurate results. The lead configuration remained unchanged throughout the plasma infusion challenge. Here is a general description of the lead placement for mouse ECG:

- Lead I: This lead measures the potential difference between the right and left forelimbs (or arms). Electrodes are placed on both the right (negative) and left forelimbs (positive). This is shown in the first line of every ECG experiment.
- Lead II: This lead measures the potential difference between the right forelimb (negative) and the left hindlimb (positive). Electrodes are placed on the right forelimb and the left hindlimb. This is shown in the second line of every ECG experiment.
- Lead III: This lead measures the potential difference between the left forelimb (negative) and the left hindlimb (positive). Electrodes are placed on the left forelimb and the left hindlimb. This is shown in the third line of every ECG experiment.
- Ground electrode: A ground electrode is placed on a neutral area, right hindlimb, to stabilize the signal and reduce noise.

Needle electrodes were connected via flexible cables to an amplifier (MicroMed, Mogliano Veneto, Italy), and then the ECG signal was recorded using System-Plus software (MicroMed, Mogliano Veneto, Italy) and sampled at 256 Hz (16 bits) with band-pass filters between 1 and 70 Hz. All animal experiments were performed without prior knowledge of the origin of the plasma samples to ensure the integrity of the results.

### Tissue preparation and immunostaining of mouse tissue

Tissue sections of the left ventricle from adult C57BL-6 mice collected in our laboratory and stored at −20°C to −80°C were cut with a cryostat (Leica CM1900), mounted on gelatin-coated histologic slides, and stored at −20°C to −80°C until use. Slides were thawed at room temperature for 10–20 min, rehydrated in wash buffer for 10 min, and incubated in an antigen unmasking solution (.01 M citrate buffer, pH 6) for 15 min at 98°C. To block non-specific binding, sections were incubated for 1 h at room temperature with 5% NDS and 5% BSA in PBS with .1% Tween 20. After washing, sections were incubated for 1 h at room temperature with patient or control plasma diluted 1:50 in PBS containing 2% NDS and 2% BSA. Sections were then washed three times for 5 min with PBS-Tween and incubated for 1 h at room temperature with anti-human IgG-FITC secondary antibody, diluted 1:200 in PBS containing 2% NDS and 2% BSA. After several washing steps, sections were incubated for 1 h with primary antibody rabbit monoclonal anti-NaV1.5, diluted 1:200 in PBS containing 2% NDS and 2% BSA, followed by washing and incubation with the appropriate anti-rabbit secondary antibody conjugated with Cy3, diluted 1:200 in PBS containing 2% NDS and 2% BSA for 1 h at room temperature. Finally, sections were washed and mounted with Vectashield mounting medium containing DAPI. Images were captured using the Leica Thunder (×40 objective).

### Human-induced pluripotent stem cells generation and culture, transcriptomic analysis, and plasma interferon-γ levels measurement

Details are reported in the Supplementary data.

### Mapping NaV1.5 epitope

A subset of 20 BrS patients was used to perform a peptide microarray–based screening for discovering putative binding epitopes of the autoantibodies on NaV1.5 protein (PEPperPRINT GmbH, Heidelberg, Germany) (see Supplementary data).

### Data analysis

Data were processed using GraphPad Prism (GraphPad Software, Inc.). Results are presented as mean ± standard deviation. Statistical significance was determined using Student’s *t*-test, analysis of variance (ANOVA), or Fisher’s exact test, depending on the nature of the data. The current–voltage relationships were tested with two-way ANOVA with Sidak *post hoc* test. A *P*-value of <.05 was deemed statistically significant.

## Results

### Study population characteristics

Fifty BrS patients (mean age 38.8 ± 12.9 years; 32 male, 64%) were enrolled in this study. Of the included patients, 15 (30%) presented a spontaneous type 1 pattern, 9 (18%) survived a previous cardiac arrest, whereas 16 (32%) experienced syncope episodes. Seven (14%) patients were found to harbour a *SCN5A* variant. A cohort of subjects with a negative SCB test served as non-BrS disease controls. Overall characteristics comparing BrS and controls are described in *Table 1* and Supplementary data online, *Table S1*. An additional cohort of patients with other cardiac diseases, channelopathy, and heart failure was also analysed (see Supplementary data online, *Table S3*).

**Table 1:** Demographic and clinical characteristics of study participants

	BrS patients (*n* = 50)	Control participants (*n* = 50)	*P*-value
Male sex, *n* (%)	32 (64)	28 (56)	.42
Age (years), mean ± SD	38.8 ± 12.9	37.6 ± 16.1	.93
Spontaneous type 1 ECG pattern, *n* (%)	15 (30)	0	<.001
Family history of sudden death, *n* (%)	26 (52)	18 (36)	.16
Family history of BrS, *n* (%)	22 (40)	18 (36)	.54
Cardiac arrest or VT/VF, *n* (%)	9 (18)	0	.003
Arrhythmic syncope, *n* (%)	16 (32)	11 (22)	.37
*SCN5A* variant, *n* (%)	7 (14)	0	.01
Anti-NaV1.5 antibodies, *n* (%)	45 (90)	3 (6)	<.001

BrS, Brugada syndrome; ECG, electrocardiogram; SD, standard deviation; VF, ventricular fibrillation; VT, ventricular tachycardia.

### Detection of anti-NaV1.5 autoantibodies in Brugada syndrome patients

**Figure 1:**
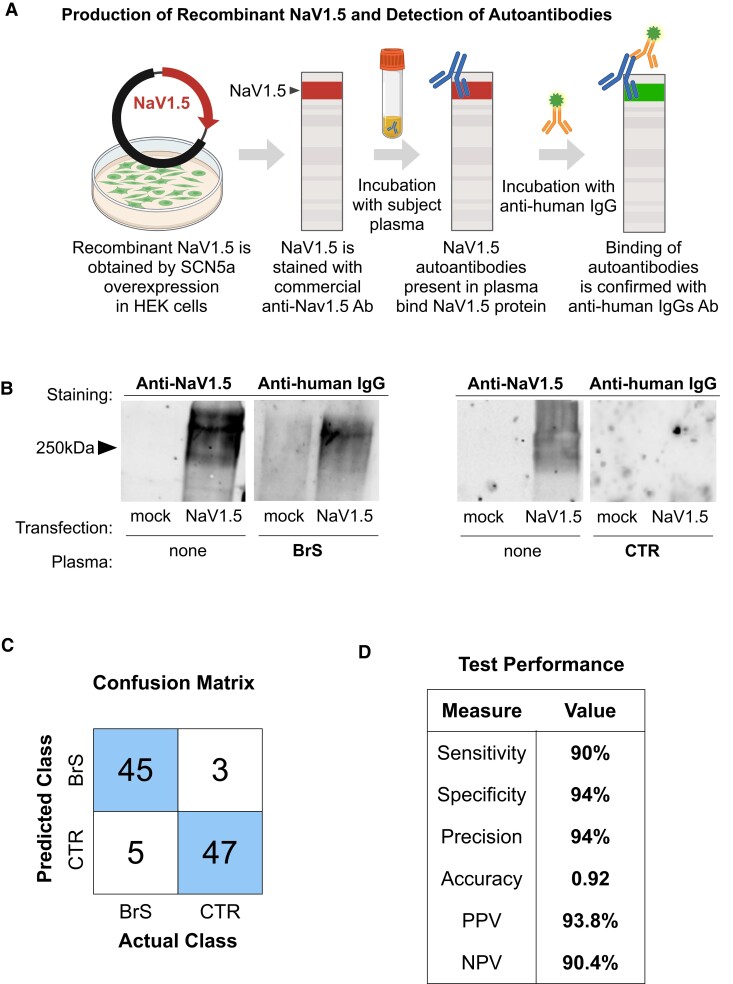
NaV1.5 targeting by plasma immunoglobulin G in Brugada syndrome. (*A*) Schematic of the workflow for detecting anti-NaV1.5 autoantibodies in Brugada syndrome. HEK293A cells over-expressing NaV1.5 are lysed, and the proteins are resolved via SDS-PAGE. The processed membrane is first probed with a commercial anti-NaV1.5 antibody, followed by incubation with plasma from subjects. Binding of plasma autoantibodies to NaV1.5 is then detected with a secondary anti-human immunoglobulin G antibody. Created with BioRender.com; (*B*) representative western blots. Left panel: anti-NaV1.5 antibody reveals the presence of the NaV1.5 protein in lysates from NaV1.5-transfected HEK293A cells but not in mock-transfected cells. Right panel: subsequent incubation with Brugada syndrome patient plasma (Brugada syndrome) and control plasma demonstrates specific binding of Brugada syndrome-derived autoantibodies to the NaV1.5 protein, as indicated by the anti-human immunoglobulin G antibody staining, absent in control plasma; (*C*) confusion matrix for the classification of BrS and control samples based on immunoglobulin G binding to NaV1.5; (*D*) table summarizing the test performance metrics derived from the confusion matrix in (*C*) measures of diagnostic efficacy, including sensitivity, specificity, precision, accuracy, and positive and negative predictive values, quantify the reliability of immunoglobulin G binding as a diagnostic marker for Brugada syndrome. BrS, Brugada syndrome; CTR, control; IgG, immunoglobulin G; NPV, negative predictive value; PPV, positive predictive value

HEK293A cells were engineered to express the NaV1.5 channel protein, and its expression was confirmed by western blot analysis using a commercial antibody specific for NaV1.5 (*Figure 1*). In the following steps, the membranes to which the protein was transferred were incubated with plasma from study participants. Binding of autoantibodies to NaV1.5 was detected with a secondary anti-human IgG antibody, as shown in *Figure 1A*. Membranes treated with plasma from BrS patients yielded a detectable signal with the anti-human IgG, indicating the presence of autoantibodies (*Figure 1B*), whereas those incubated with plasma from control subjects and patients suffering from channelopathy, heart failure, and other cardiac diseases did not exhibit such binding, as shown in Supplementary data online, *Figures S1* and *S2*. To verify that IgG from the plasma of BrS patients specifically binds to the NaV1.5 channel, two key experiments were performed. First, patient plasma was applied directly to the membranes without prior staining with an antibody to exclude any influence of prior application of a commercial anti-NaV1.5 antibody on our results. This direct approach confirmed that the IgG from the plasma still specifically targeted the NaV1.5 protein, as shown in Supplementary data online, *Figure S3A* and *B*. For the second experiment, to denatured IgGs from plasma of BrS patients, samples were heated to 100°C for 10 min and then applied on the membranes. This process confirmed that once denatured, the IgGs could no longer specifically bind to the NaV1.5 channel, as evidenced by the absence of signal in the western blot (see Supplementary data online, *Figure S3C* and *D*). In this study, anti-NaV1.5 antibodies were detected in 90% of patients diagnosed with BrS, a significant difference from the 6% found in the control group. It was found that the clinical phenotype of BrS patients did not differ significantly between those with and without detectable levels of anti-NaV1.5 IgGs, as shown in Supplementary data online, *Table S4*. The diagnostic efficacy of antibody detection was notable, with a specificity of 94% and a sensitivity of 90%, yielding a positive predictive value (PPV) of 93.75% and a negative predictive value (NPV) of 90.38%, as shown in *Figure 1C* and *D*. The study was extended to whole-exome sequencing. This analysis revealed that autoantibodies against NaV1.5 were detected in many patients, irrespective of their *SCN5A* mutation status, age, gender, or the presence of the characteristic type 1 BrS ECG pattern (see *Table 1* and Supplementary data online, *Table S1* for further details). The presence of anti-NaV1.5 IgGs was also investigated in a cohort of 35 patients with other cardiac diseases, including channelopathies such as long QT syndrome, heart failure, and cardiomyopathies (see Supplementary data online, *Table S3*). IgGs against NaV1.5 could not be detected in any of these cardiac patients by western blot analysis (see Supplementary data online, *Figure S2*). Immunoprecipitation assays were also performed using protein lysates from HEK293A cells over-expressing the NaV1.5 channel and from mouse heart tissue. These assays were designed to investigate whether IgGs from BrS patients can specifically bind to the NaV1.5 protein in its natural, folded conformation. The methods and results of these assays are described in detail in the Supplementary data and in Supplementary data online, *Figure S4*.

### Determination of the specificity of anti-NaV1.5 autoantibodies

**Figure 2:**
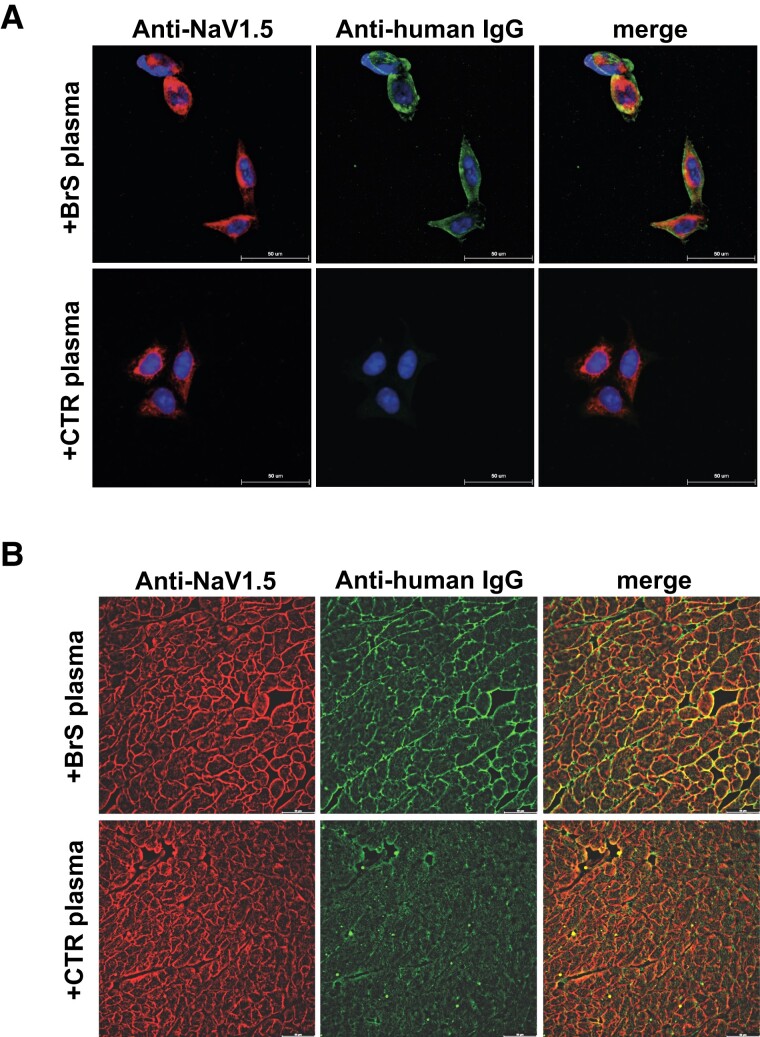
Validation of anti-NaV1.5 autoantibody specificity and diagnostic efficacy in Brugada syndrome. (*A*) Immunofluorescence assays on HEK293A cells over-expressing NaV1.5. Top: Cells treated with plasma from Brugada syndrome patients show specific binding of immunoglobulin Gs (green) to the NaV1.5 channel (red). Bottom: No immunoglobulin G binding is observed in cells treated with control plasma, indicating specificity of the antibody–channel interaction; (*B*) tissue immunofluorescence demonstrating NaV1.5 channel localization in mouse heart sections. Top: Treatment with Brugada syndrome patient plasma (+Brugada syndrome plasma) results in the co-localization of human immunoglobulin Gs (green) with NaV1.5 expression (red), indicating autoantibody binding to the channel. Bottom: Control plasma (+control plasma) shows no such co-localization, underscoring the specificity of the Brugada syndrome patient autoantibodies. BrS, Brugada syndrome; CTR, control; IgG, immunoglobulin G

To test the specificity of the autoantibodies against NaV1.5, immunolabelling assays were performed using HEK293A cells engineered to over-express NaV1.5. In these assays, commercially available anti-NaV1.5 antibodies and plasma from study participants were used as primary antibodies. The results showed co-localization where the commercial NaV1.5 antibody (red) overlapped with the autoantibodies detected in green, but only in cells treated with plasma from BrS patients (*Figure 2A*). This co-localization was not present in cells treated with control plasma, indicating specific binding of the BrS autoantibodies to NaV1.5 (*Figure 2A*). In contrast, when this experiment was repeated with MOCK-HEK293A cells not expressing NaV1.5, no fluorescent signal was detected regardless of the plasma source (see Supplementary data online, *Figure S5*). Further studies were performed with heart tissue samples from mice expressing a NaV1.5 isoform that shows significant homology (93%) with the human isoform. These samples were incubated with either control or BrS patient plasma. Immunostaining with the commercial NaV1.5 antibody (red) and detection of IgGs (green) in these tissues also showed co-localization exclusively in the samples treated with BrS plasma (*Figure 2B*). This was not observed in the control plasma-treated samples, highlighting the specificity of the BrS autoantibodies for NaV1.5 (*Figure 2B*).

To further confirm the specific binding of IgGs from BrS patients to the NaV1.5 channel, epitope mapping was outsourced to PEPperPRINT (Heidelberg, Germany) using their high-density peptide array technology (see Supplementary data). The assay focused on the extracellular loops of NaV1.5 and translated these regions into overlapping peptides that were visualized on the microarrays. Analysis of plasma samples from a subset of 20 BrS patients (see Supplementary data online, *Table S2*) revealed binding to specific segments of the extracellular loops of NaV1.5 (see Supplementary data and Supplementary data online, *Figure S6*). An additional cohort of 35 patients, diagnosed with other cardiac conditions served as non-BrS controls to test the disease-related autoantibodies specificity. Western blot analysis presented in Supplementary data online, *Figure S2* showed the absence of NaV1.5 autoantibodies in this cohort.

### Effects of NaV1.5 autoantibodies on sodium currents *in vitro*

**Figure 3:**
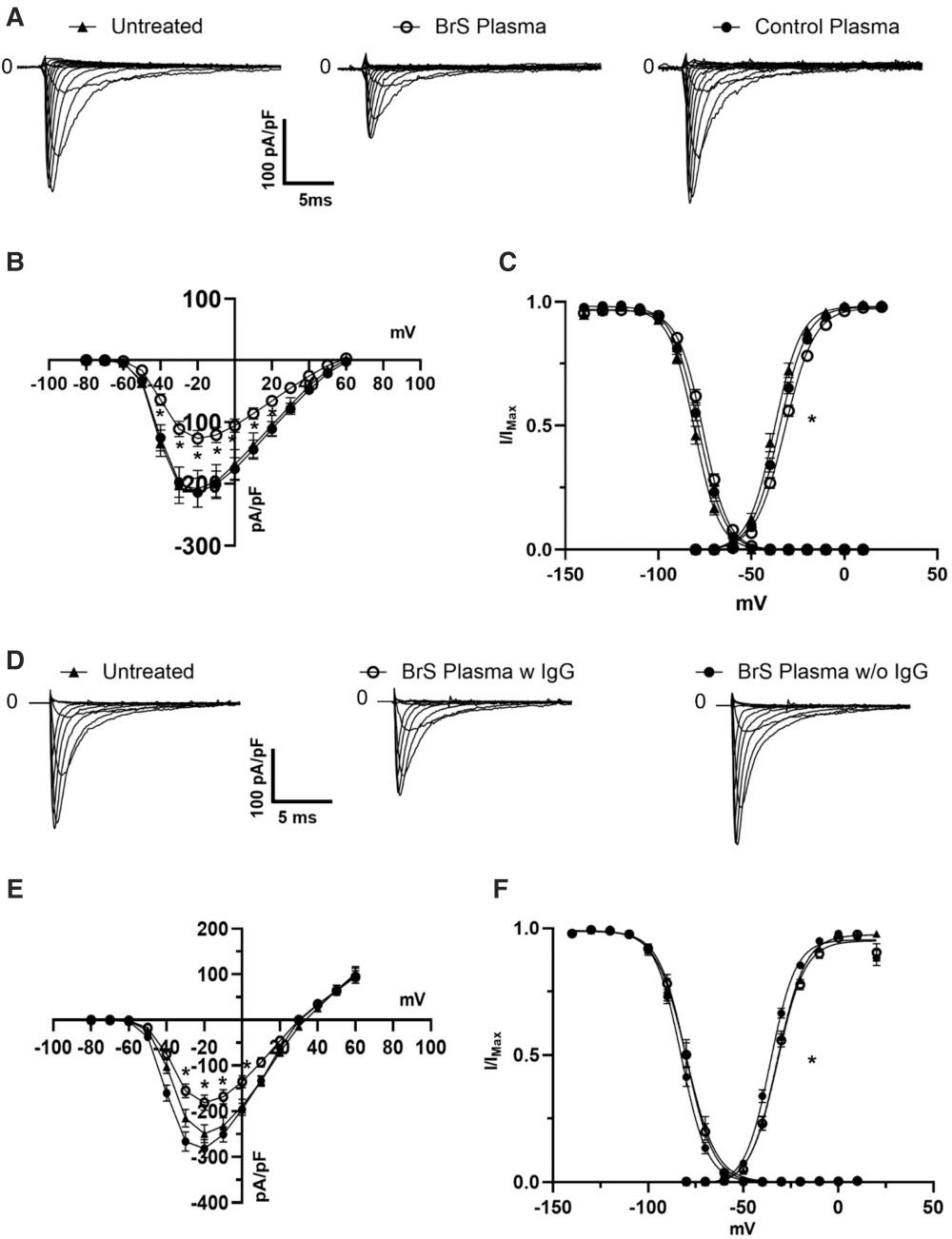
Impact of Brugada syndrome patient autoantibodies on NaV1.5 sodium current and channel localization. (*A*) Representative traces of sodium currents recorded from HEK293A cells over-expressing NaV1.5 untreated (left) or after incubation with Brugada syndrome patient plasma (centre) vs. control plasma (right). The Brugada syndrome panel demonstrates reduced current amplitudes compared with the control panel; (*B*) current–voltage relationship (I–V curve) of NaV1.5 currents. Data from Brugada syndrome plasma- (*n* = 70, *N* = 6, empty circles) and control plasma-treated cells (*n* = 91, *N* = 8, filled circles) reveal a marked decrease in peak sodium current in the presence of Brugada syndrome plasma, indicating functional modulation of the channel; untreated cells are in black triangle (*n* = 35, *N* = 7); (*C*) normalized conductance–voltage relationships highlight a shift in the voltage dependence of activation, with Brugada syndrome plasma-treated cells (filled circles) displaying altered channel kinetics compared with controls (empty circles) and untreated cells (filled triangles); (*D*) families of sodium current elicited in untreated cells (left) or in cells incubated with complete Brugada syndrome plasma (centre) or with BrS plasma devoid of immunoglobulin G (right); (*E*) I–V curve of the three condition tested: untreated (filled triangles, *N* = 3, *n* = 24), Brugada syndrome complete plasma (empty circles, *N* = 3, *n* = 25), and Brugada syndrome plasma devoid of immunoglobulin G (black circles, *N* = 3, *n* = 36); (*F*) voltage dependence of channel activation and inactivation (*P < .5). For values, see Supplementary data. BrS, Brugada syndrome; IgG, immunoglobulin G

To evaluate the impact of autoantibodies on NaV1.5, HEK293A cells expressing NaV1.5 were exposed to BrS plasma concentrations ranging from .5% to 10%. MTT assays determined that 5% was the highest non-toxic concentration (see Supplementary data online, *Figure S7A*). Patch-clamp experiments revealed that a 1-h incubation with 5% BrS patient plasma exhibited a significant reduction in current density (approximately 40%) compared with cells incubated with plasma from control subjects (*Figure 3*). The mean peak current density after a depolarizing voltage step at −20 mV was −122.3 ± 12 pA/pF (*n* = 91) with plasma from BrS patients, in contrast to −211.2 ± 21 pA/pF (*n* = 70) from controls (*Figure 3A–C*). A Boltzmann fit of experimental data yielded *V*_1/2_ values of −31.3 ± .4 mV (*k* = 8.4 ± .4) and −34.4 ± .4 mV (*k* = 7.7 ± .3) for BrS patient and control plasma, respectively. It should be noted that the observed shift in voltage dependence of activation, although statistically significant, is apparently not sufficient to explain the substantial reduction in sodium current (*Figure 3C*). No significant alterations were observed in the voltage dependence of steady-state inactivation with a *V*_1/2_ value of −76.8 ± .4 mV (*k* = 7.3 ± .4) in the presence of the BrS plasma and −78.9 ± .3 mV (*k* = 7.3 ± .2) in control plasma. This effect was dose dependent (see Supplementary data online, *Figure S7B*) and caused by the presence of IgG (*Figure 3D–F*; Supplementary data online, *Table S5*) and was specific for the sodium current (see Supplementary data online, *Figures S8*–*S10* and *Table S5*–*S8*). Along this line, the reduction observed could be explained *in vitro* by the loss of the content of NaV1.5 protein channel on the cell membrane, as shown in Supplementary data online, *Figure 1^1^*.

### Effects of Brugada syndrome patient plasma injection in wild-type mice

**Figure 4:**
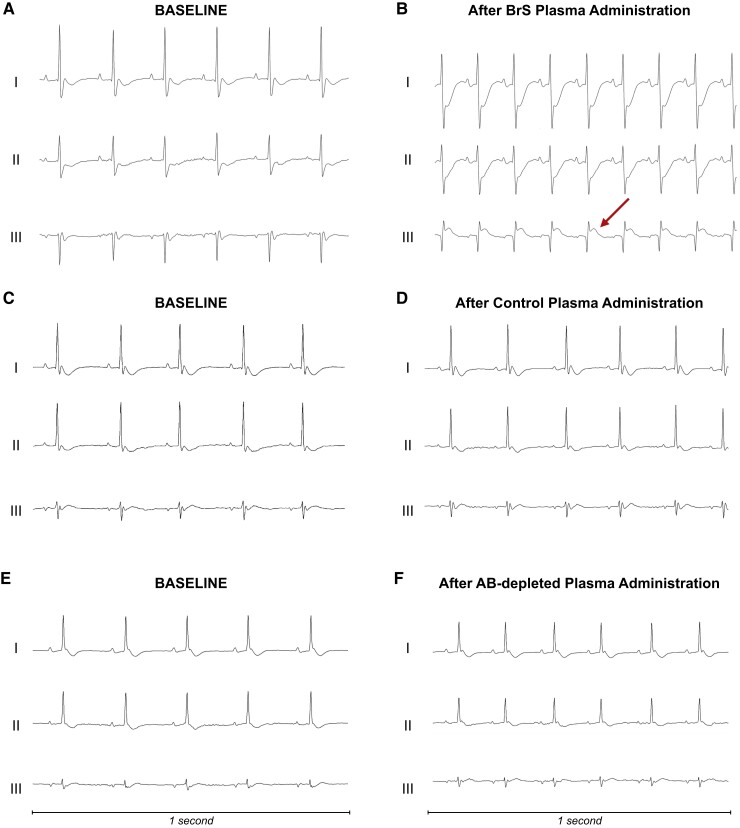
Electrocardiographic response to plasma *in vivo*. (*A*, *B*) The electrocardiographic recordings from a mouse before (*A*) and after (*B*) receiving plasma from a patient with Brugada syndrome. Following the administration, the electrocardiographic mirrors the Brugada-like electrocardiographic pattern commonly observed in humans in bipolar leads, indicated by ST-segment elevation in Lead III and specular ST-segment depression in Leads I and II. This mouse died after the experiment and all the electrocardiographic changes developing after the infusion are shown in detail in Supplementary data. (*C*, *D*) The electrocardiographic outcomes from a mouse treated with plasma from a control subject without Brugada syndrome. Notably, no electrocardiographic changes are evident following the infusion (*D*), indicating the absence of arrhythmogenic activity. (*E*, *F*) Electrocardiographic tracings from a mouse receiving antibody-depleted Brugada syndrome plasma. No electrocardiographic abnormalities could be demonstrated following the infusion. Three bipolar lead configurations for electrocardiographic recording are shown (Leads I, II, and III, respectively). Further electrocardiographic details are shown in Supplementary data. AB, antibody; BrS, Brugada syndrome

Continuous ECG monitoring of seven mice expressing the wild-type isoform of NaV1.5 channels that were intravenously administered plasma from four BrS patients and three control subjects under general anaesthesia showed that the BrS plasma-infused mice developed Brugada ECG ST-segment abnormalities (*Figure 4* and Supplementary data online, *Figures S12*–*S18*). Mouse #1 showed a Brugada-like pattern followed by complex malignant arrhythmias (ventricular arrhythmias and AV block) leading to asystole and death (*Figure 4A* and *B* and Supplementary data online, *Figure 1^2^*). Mouse #2 showed transient complete AV block with coved-type ST-segment elevation (see Supplementary data online, *Figure 1^3^*). Mouse #3 demonstrated transient severe malignant ventricular arrhythmias (see Supplementary data online, *Figure 1^4^*) and a Brugada-like ECG pattern (see Supplementary data online, *Figures S14* and *S19*). In a further experiment, a fourth mouse was infused with antibody-depleted plasma from the same BrS patient previously used for Mouse #3, whose administration resulted in coved-type ST-segment elevation pattern and malignant arrhythmic phenotype. Of note, this administration did not result in any ECG changes (see Supplementary data online, *Figure S18*). Moreover, mice receiving control plasma exhibited no ECG alterations, including ST-segment or conduction disturbances (*Figure 4C* and *D* and Supplementary data online, *Figures S15*–*S17*). All detailed ECG tracings are included in the Supplementary data.

### Transcriptomic analysis of human peripheral blood mononuclear cells (PBMCs) in Brugada syndrome patients

**Figure 5:**
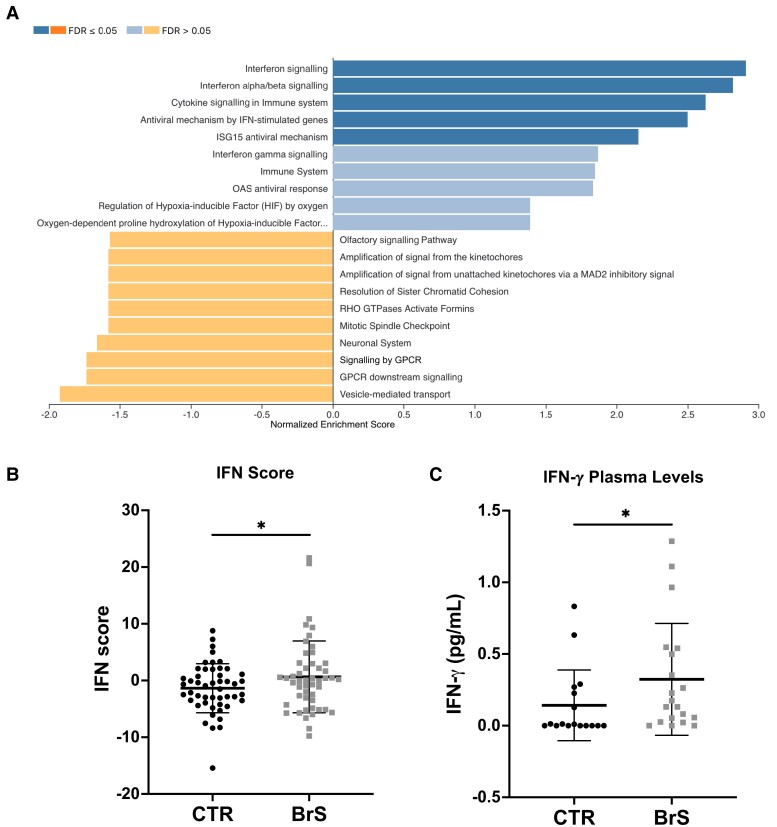
Transcriptomic and immunologic changes in Brugada syndrome patients. (*A*) Gene Set Enrichment Analysis: Transcriptomic analysis of PBMCs of Brugada syndrome patients compared with healthy controls shows 540 differentially expressed genes. Gene Set Enrichment Analysis shows significant up-regulation of immune signalling pathways, especially those related to cytokine and interferon signalling. (*B*) Interferon score: A composite interferon score, calculated as the sum of *Z*-scores for nine major interferon-related genes, is significantly elevated in Brugada syndrome patients, indicating increased activity of the interferon signalling pathway. (*C*) Interferon-γ plasma concentrations: Measurement of plasma levels of interferon-γ by enzyme-linked immunosorbent assay further confirms this up-regulation, showing significantly higher interferon-γ levels in Brugada syndrome patients compared with controls. Data were analysed with student *t*-test (*P < .05). BrS, Brugada syndrome; CTR, control; IFN, interferon

Transcriptomic analysis of PBMCs in our study cohort was performed including both BrS patients and healthy controls. This analysis identified 540 genes with differential expression in BrS patients showing a distinct transcriptomic profile (false discovery rate ≤ .05; *Figure 5*). Evaluation of the enrichment of these differentially expressed genes revealed significant up-regulation in immune signalling pathways, with a focus on cytokine and interferon (IFN) signalling pathways (*Figure 5A*). To quantify the involvement of the IFN signalling pathway, IFN-γ score was calculated. This score, derived from the expression levels of nine IFN-related genes, was significantly higher in BrS patients compared with controls (*Figure 5B*). This increase in IFN score indicates an increased activity of the IFN signalling pathway in BrS. To further corroborate these transcriptomic findings, the plasma levels of IFN-γ were measured through an enzymatic immunoassay enzyme-linked immunosorbent assay (ELISA). The results showed a significant increase in IFN-γ levels in BrS patients (*Figure 5C*), confirming the activation of the IFN signalling pathway observed in the transcriptomic analysis.

## Discussion

This study reveals the presence of autoantibodies against the NaV1.5 sodium channel in patients with BrS, supporting their dual utility as diagnostic markers and potential effectors in the pathogenesis of the disease (*Structured Graphical Abstract*).

The conventional hallmark for BrS diagnosis has been the type 1 ECG pattern, which may be transient and difficult to capture.^2^ The discovery that these autoantibodies are present in a significant majority (90%) of BrS patients, whereas they are rare in control subjects (6%), confirms their potential as a distinctive marker for BrS and may provide an additional diagnostic tool to complement existing ECG criteria and reduce reliance on provocation testing. The presence of anti-NaV1.5 IgGs was independent of factors such as *SCN5A* mutation status, age, gender, or ECG pattern of the patients, and it suggests a broader underlying pathophysiological mechanism of BrS. It is noteworthy that the specificity of these autoantibodies for BrS was supported by their absence in the plasma of a cohort of patients affected by other cardiac pathologies.

Remarkably, the infusion of BrS patient plasma into wild-type mice not only induced severe arrhythmic events but also, for the first time, replicated the distinctive coved-type ST-segment elevation seen in humans. Of note, these effects were not observed in mice injected with either control plasma or BrS plasma depleted of IgGs, directly linking autoantibodies to the development of BrS phenotype. Remarkably, the ECG abnormalities documented in the murine model clearly resemble those seen in humans in the bipolar leads.^34,35^ In fact, while the typical type 1 pattern is traditionally observed in right-sided unipolar leads (i.e. V1 and V2), a significant proportion of patients with a prominent spontaneous type 1 BrS pattern also show reciprocal changes as ascending ST-depression in bipolar leads.^36^ Similar abnormalities were observed also in our mouse model, further supporting the role of the autoantibodies in eliciting a Brugada-like ECG phenotype.

*In vitro* experiments with HEK293A cells over-expressing NaV1.5 and cardiomyocytes derived from induced pluripotent stem cells (iPSCs) showed a reduction in sodium current density when exposed to BrS patient plasma, providing additional insight into the mechanism. Furthermore, the exclusive effect on sodium current density as opposed to calcium currents suggests a selective pathogenic mechanism. These findings are consistent with previous studies linking autoantibodies to altered ion channel function in other cardiac diseases.^29,37–39^ Moreover, BrS patient autoantibodies could trigger NaV1.5 channel internalization, further suggesting a new pathogenic mechanism. This theory is supported by research indicating that autoantibodies targeting membrane proteins can alter the ionic current by triggering internalization of membrane channels, as observed in our cohort.^40–43^ Taken together, these results support a role for these autoantibodies in modulating the behaviour of the NaV1.5 channel, resulting in the electrophysiological abnormalities characteristic of BrS, including coved-type ST-segment elevation, life-threatening ventricular arrhythmias, and severe conduction disturbances. Other studies outside of BrS have also highlighted the immunogenic properties of the NaV1.5 channel.^44^ Although the authors focused on the conduction abnormalities caused by reduced *I*_Na_ induced by autoantibodies against the channel, the J-wave abnormalities reported in the study closely resemble those reported in a heterozygous knock-in mouse model of BrS (*SCN5A*G1746R/+).^45^ Nevertheless, they may explain previous findings showing NaV1.5 aggregates in post-mortem heart samples from BrS patients, which could potentially trigger the observed immune response.^23^ While our results align with sodium channel blockade as the primary mechanism for the electrophysiological abnormalities observed in our mouse model, similar to findings in humans,^46–48^ an imbalance in the autonomic nervous system may also contribute. Nav1.5 is present in other heart cell types, such as ganglia, where its dysfunction could lead to extracardiac effects exacerbating cardiac function and arrhythmias.^49^ This might provide a potential concurrent mechanistic influence that calls for further exploration. Transcriptomic analysis of PBMCs from BrS patients further supports the findings of this study by showing altered gene expression in immune signalling pathways. In particular, the significant up-regulation of cytokine and IFN signalling pathways and the increased IFN-γ levels in plasma provide molecular evidence for an active immune component in BrS. This complements the observation of autoantibody-mediated effects on the NaV1.5 channel and suggests a broader involvement of the immune system in the pathogenesis of the disease. While these findings are compelling, they require further in-depth mechanistic investigation. Nevertheless, both *in vitro* and *in vivo* results show that binding of autoantibodies leads to decreased sodium currents, i.e. reduced *channel reserve*, which may explain why SCBs can induce the type 1 ECG pattern in all patients, regardless of *SCN5A* mutation status. This autoimmune mechanism could also support the very dynamic nature of the epicardial substrate that is influenced by various factors.^50,51^

The evolving field of immunotherapy for cardiovascular disease holds promise for similar approaches in BrS. For instance, the CANTOS trial demonstrated the efficacy of targeting inflammation in atherosclerosis with an anti-interleukin-1β antibody and found a reduction in cardiovascular events.^52^ In addition, the use of mepolizumab, an antibody against interleukin-5, in eosinophilic myocarditis demonstrates the feasibility of targeted immunotherapies.^53^ However, patient response variability and potential side effects call for careful development and optimization of these therapies. Moreover, advances in more sensitive detection methods may allow correlation of autoantibody levels with disease severity and progression in BrS. Most importantly, the quantification of these autoantibodies could serve as a tool for evaluating the efficacy of BrS treatments such as epicardial ablation.^54,55^ In this way, it would be possible to determine whether such interventions lead to substantial molecular changes or merely a reduction in phenotypic burden, as evidenced by the normalization of the ECG. This approach could open new avenues to assess the true therapeutic impact of current and future treatments for BrS.

### Limitations

The detection of autoantibodies in most BrS patients underscores their diagnostic value. However, caution is needed due to our cohort’s limited size. These findings lay the groundwork for larger, multi-centre studies to validate the clinical utility of autoantibody testing. The western blot method's sensitivity limits might miss extremely low autoantibody titres in some patients. This issue could be addressed by developing more sensitive tests, such as a diagnostic ELISA, to enhance detection and clinical relevance. Still, our autoantibody detection's sensitivity and specificity are on par with other cardiology tests, such as exercise electrocardiography, and outperform other medical screening tests, such as B-type natriuretic peptide for heart failure [Area under curve (AUC) .60–.70],^56^ Papanicolaou smear for cervical cancer (AUC .70),^57^ and the CHA_2_DS_2_-VASc score for stroke risk (AUC .57–.72).^58^ Clinicians should consider the prevalence of BrS in real-world settings when interpreting the assay results. While the NPV would remain very high, the PPV may be affected by the higher ratio of negative subjects in the general population. Another limitation was the inability to obtain cardiac tissue samples from BrS patients, primarily due to the high-risk nature of such biopsies. Nonetheless, even with access to these samples, detecting NaV1.5-specific autoantibodies would have posed a significant challenge. This difficulty stems from the multitude of different IgGs naturally present in cardiac tissue.^37^ To address this, a technique used in identifying human brain protein autoantibodies was adapted in this study.^59^ Mouse heart tissue was exposed to plasma from BrS patients and control subjects and the presence of human autoantibodies against NaV1.5 was detected with a specific stain. Human IgGs directed against NaV1.5 were detected in the samples from BrS patients, which was not the case in the control samples.

## Conclusions

This study expands our understanding of BrS by demonstrating the presence of anti-NaV1.5 autoantibodies in patients. This introduces an immune-mediated component to BrS pathogenesis, independent of *SCN5A* mutation status and ECG patterns. Autoantibody testing could significantly improve BrS diagnostic accuracy. Future research should aim to unravel the exact role of these autoantibodies in BrS pathophysiology and explore their potential in developing targeted therapies, especially in genetically elusive BrS cases.

## Supplementary Material

ehae480_Supplementary_Data

## Declarations

### Data Availability

The data underlying this article are available in the article and in its online supplementary material.

### Pre-registered Clinical Trial Number

None supplied.
