# Supplementary material for: NaV1.5 autoantibodies in Brugada syndrome: pathogenetic implications
Source: Eur Heart J. 2024 Jul 30;45(40):4336–48. doi: 10.1093/eurheartj/ehae480 (PMC11491155; doi:10.1093/eurheartj/ehae480)
Supplement: ehae480_Supplementary_Data [file ehae480_supplementary_data.zip › Supplementary_data_Rev3 add exp_PL.pdf]

Supplementary data

NaV1.5 Autoantibodies in Brugada Syndrome: Pathogenetic Implications

Adriana Tarantino[1,2,†], Giuseppe Ciconte[1,2,3,†], Dario Melgari[1], Anthony Frosio[1], Andrea Ghiroldi[1], Marco Piccoli[1], Marco Villa[1], Pasquale Creo[1], Serena Calamaio[1], Valerio Castoldi[4], Simona Coviello[1], Emanuele Micaglio[1,3], Federica Cirillo[1], Emanuela Teresina Locati[1,3], Gabriele Negro[1,3], Antonio Boccellino[3], Flavio Mastrocinque[1,3], Žarko Ćalović[3], Stefano Ricagno[1,5], Letizia Leocani[2,4], Gabriele Vicedomini[1,3], Vincenzo Santinelli[3], Ilaria Rivolta[1,6], Luigi Anastasia[1,2,#,*], and Carlo Pappone[1,2,3,#,*]

[†]These authors contributed equally to this work and share first authorship. [#]These authors contributed equally to this work and share last authorship.

[1]Institute for Molecular and Translational Cardiology (IMTC), IRCCS Policlinico San Donato, piazza Malan 2, 20097, San Donato Milanese, Milan, Italy;

[2]School of Medicine, University Vita-Salute San Raffaele, via Olgettina 58, 20132, Milan, Italy;

[3]Arrhythmology Department, IRCCS Policlinico San Donato, piazza Malan 2, 20097, San Donato Milanese, Milan, Italy;

[4]Experimental Neurophysiology Unit, Institute of Experimental Neurology-INSPE, IRCCS Ospedale San Raffaele, via Olgettina 58, 20132, Milan, Italy

[5]Department of Biosciences, Università degli Studi di Milano, Milan 20133, Italy;

[6]School of Medicine and Surgery, University of Milano-Bicocca, Via Cadore, 48, 20900 Monza, Italy

[*]To whom correspondence may be addressed: anastasia.luigi@hsr.it Tel.: +39 (02) 2643-7756 and carlo.pappone@af-ablation.org Tel.: +39 (02) 5277-4260/4306

Table of contents

Supplementary Fig. 1. Pag 12
Supplementary Fig. 2. Pag 18
Supplementary Fig. 3. Pag 19
Supplementary Fig. 4. Pag 20
Supplementary Fig. 5. Pag 22
Supplementary Fig. 6. Pag 23
Supplementary Fig. 7. Pag 25
Supplementary Fig. 8. Pag 26
Supplementary Fig. 9. Pag 28
Supplementary Fig. 10. Pag 30
Supplementary Fig. 11. Pag 32
Supplementary Fig. 12. Pag 34
Supplementary Fig. 13. Pag 37
Supplementary Fig. 14. Pag 39
Supplementary Fig. 15. Pag 41
Supplementary Fig. 16. Pag 42
Supplementary Fig. 17. Pag 43
Supplementary Fig. 18. Pag 44
Supplementary Fig. 19. Pag 45
Supplementary Table. 1. Pag 47
Supplementary Table. 2. Pag 48
Supplementary Table. 3. Pag 49
Supplementary Table. 4. Pag 51
Supplementary Table. 5. Pag 52
Supplementary Table. 6. Pag 52
Supplementary Table. 7. Pag 53
Supplementary Table. 8. Pag 54
References Pag 55

Supplementary Methods

Study Population

Patients in the BrS were diagnosed according to the current consensus guidelines[1]. BrS was diagnosed in the presence of a coved-type ST elevation of ≥2mm as documented in ≥1 lead from V1 to V3 positioned in the second, third, or fourth intercostal space. Because of the variable nature of the BrS-ECG pattern, BrS patients were classified according to their ECG at the time of the presentation and defined as spontaneous ECG type 1 pattern. ECG pattern definitions were based on the second consensus statement regarding BrS [2].

All the clinical characteristics of the BrS patients are detailed in Table 1, including presence of spontaneous type 1 ECG pattern, prior symptoms including aborted cardiac arrest or syncope, family history of BrS or sudden death, and *SCN5A* variants.

List of inclusion and exclusion criteria are the following and have been included in the main manuscript:

BrS group, Inclusion criteria:

- Patients affected by BrS diagnosed according to current guidelines document criteria
- Age > 18
- Willingness to participate in the study analysis

Control Group, Inclusion criteria:

- Age > 18
- Rule out of BrS diagnosis by the means of a sodium channel blocker challenge

Exclusion criteria, valid for both study groups:

- Documented cardiomyopathy or structural cardiac abnormalities
- Pregnancy or breast-feeding,
- Life expectancy < 12 months.
- Age ≤18

Whole Exome Sequencing

A subset of BrS patients (n=37) was screened for *SCN5A* gene mutations using Whole Exome Sequencing. Genomic DNA was extracted from 0.3 ml of fresh blood samples using the Maxwell® RSC Blood DNA Kit,

following the manufacturer's instructions. DNA concentration was measured with the Qubit Fluorometer (Thermo Fisher Scientific). DNA libraries were prepared following the TruSeq Exome Library Prep protocol (Illumina), starting with 50 ng of DNA. Libraries were barcoded, pooled, and sequenced on an Illumina NextSeq 2000, yielding 100 M paired end reads (100 bp). The sequencing data were processed using the DRAGEN Enrichment pipeline (version 3.8.4), referencing the Ensembl 98 Human Genome build (GRCh38.p13). *SCN5A*-specific variant analysis was conducted using Illumina's Variant Interpreter Software.

NaV1.5 Immunoprecipitation

HEK293A cells expressing NaV1.5 were lysed as described in the Methods. BrS patient and healthy control IgGs, extracted from plasma using Dynabeads™ Protein G (Thermo Fisher Scientific) according to the datasheet, were incubated with 300 ug of protein lysate for 2 h at room temperature with orbital shaking after two washes with TBS. The beads were washed three times with TBS, and Laemmli-β-mercaptoethanol buffer was added for a 5-minute incubation at 100 °C. The eluted fraction was subsequently subjected to western Blot for NaV1.5 as described in the Methods. To collect heart samples for NaV1.5 immunoprecipitation, adult mice C57BL-6 were anesthetized by intraperitoneal injection of medetomidine, 0.5 mg/kg (Orion Pharma S.r.l.) and ketamine, 100 mg/kg (Merial), both diluted in saline solution. Once the animals were completely unconscious, their thorax was opened, and the heart was perfused with 1 ml KCl 1M to induce diastolic arrest and flushed with 0.9% saline through a cannula inserted into the left ventricle. The heart was then excised, and the left ventricle was separated from the atria and right ventricle. The left ventricle was then divided into 3-mm-thick slices using a specific stainless steel heart matrix (Roboz Surgical Instruments) to create apex, middle, and base specimens. The apex was incubated with 500 ml RIPA Lysis Buffer and homogenized using the tissue homogenizer Lyser® (Qiagen). Each sample was subjected to three homogenization cycles, each lasting 5 minutes at a rate of 25 oscillations per second. The homogenate was kept in ice for 30 minutes and then centrifuged at 10000x*g* for 10 minutes at 4 °C. After centrifugation, the supernatant of each tissue sample was transferred to a new tube, and the total protein content was quantified using the BCA Protein Assay Kit (Thermo Fisher Scientific) according to the manufacturer’s instructions.

300 ug of total protein were incubated with Dynabeads™ Protein G bound with IgGs from BrS plasma or control, and the eluted fraction was subsequently subjected to western blot analysis stained for NaV1.5, as described in the Methods.

hiPSC culture and in vitro cardiac differentiation

An hiPSC line from a healthy female donor (obtained from Thermo Fisher Scientific, cell line: TMOi001-A) was used and maintained on human Biolaminin 521 LN-coated dishes in TeSR-E8 TM medium (Thermo Fisher)[3]. Cardiac differentiation was conducted through monolayer culture on Matrigel® hESC-qualified Matrix (Corning, Corning, NY, USA) dishes using the PSC Cardiomyocytes Differentiation Kit (Thermo Fisher Scientific), following manufacturer instructions. On the 21st day of differentiation, human induced Pluripotent Stem Cells-Derived Cardiomyocytes (hiPSC-CMs) cultures were enriched using the PSC-Derived Cardiomyocyte Isolation Kit (Miltenyi Biotech) and maintained until day 28 in culture in Cardiomyocytes Maintenance Medium (CMM) with B27 supplement (Thermo Fisher). For electrophysiological experiments, hiPSC-CMs were detached, and single cells were replated on Matrigel-coated 35mm dishes (VWR) for at least 48 h before conducting experiments. hiPSC-CMs were then incubated, either with or without 5% plasma from BrS patients, for 1 h at 37°C, 5% CO_2.

MTT assay cell viability

A colorimetric assay based on tetrazolium salt MTT (3-(4,5-dimethylthiazol-2-yl)-2,5-diphenyl tetrazolium bromide), was performed to evaluate the viability of cells upon the plasma treatment. Briefly, HEK293 cells expressing NaV1.5 channel were cultured and exposed to 0.05, 5 and 10% of pre-heated plasma in transparent/clear flat bottom 96-wells plates for 1h followed by incubation with 0.5 mg/ml MTT for 1 h at 37°C and 5% (v/v) CO_2. Then the medium was removed and formed formazan crystals were dissolved in DMSO and ethanol (1:1 diluted) on a microplate shaker for 20 min at room temperature. Absorption was measured at 570 nm and subtracted from background at 670 nm (Supplementary Fig. 7).

Evaluation of dose-response effect on NaV1.5 sodium current

The dose-response effect of BrS plasma was evaluated comparing the fractional inhibition of the sodium current density in HEK293Acells overexpressing NaV1.5incubated for 1 h in the presence of 3 different BrS plasma at 5% or 0.5% concentration. Higher concentrations were excluded as already at10% the plasma tended to gelatinize, affecting cellular viability.

Surface protein biotinylation

HEK293A cells over-expressing NaV1.5 protein were treated with patients or controls' plasma for 1 h, then washed twice with PBS, and incubated with 0.5 mg/ml EZ Link Sulfo-NHS-LC Biotin (Pierce) in cold PBS for 20 minutes at 4 °C. The excess of free biotin was quenched by incubating with Glycine 100 mM for 5 minutes at 4 °C. Cells were rinsed in PBS and lysate with RIPA lysis buffer, previously described for 30 minutes at 4 °C. Cells debris were removed by centrifugation at 15,000 rpm for 10 minutes, the supernatant was collected, and protein concentration measured using the BCA assay. Equal amounts of biotinylated proteins were incubated with avidin-linked agarose beads at 4 °C overnight. Beads were washed three times with PBS and protein elution was performed in Laemmli-b-mercaptoethanol (Bio-Rad) for 5 min at 100°C. Immunoprecipitated fraction was resolved by western blot as previously described. The quantification of NaV1.5 membrane protein loss was assessed by calculating the ratio relative to the total membrane protein content. Densitometric analysis of the western blots was performed with Image Lab software (Bio-Rad).

Current measured in hiPSC-CM

Inward currents were elicited by manual patch-clamp at 37°C in whole-cell configuration applying a step protocol from -80 to 60 mV, 150 ms duration (holding -80mV). The protocol was applied in absence of any drugs, in presence of 10 μM nifedipine, and in presence of 10 μM nifedipine and 30 μM TTX. Nifedipine-sensitive I_{CaL} and TTX-sensitive I_{Na} were obtained by subsequent subtraction during the analysis. Intracellular solution was (mM): 135 CsCl, 10 NaCl, 5 EGTA, 2$CaCl_2$, 2 TEA-Cl, 10 HEPES, 2 MgATP; pH 7.2 with CsOH. In the extracellular solution NaCl was 80 mM.

Cav 3.2 calcium channel currents

HEK293T overexpressing Cav3.2 were obtained by B'SYS GmbH. For automated patch-clamp experiments, intracellular solution was (mM): CsCl 10, CsF 110, NaCl 10, EGTA 10, TEA-Cl 2, HEPES 10; pH 7.2 CsOH; while the extracellular (mM): TEA-Cl 120, CsCl 10, $CaCl_2$ 2, MgCl2 1, HEPES 10, Glucose 20; pH 7.4 CsOH [4]. The currents were elicited by a depolarizing voltage steps protocol (holding -120 mV), from -80 to 60 mV of 500 ms duration, and the inactivation was studied applying a 2-steps protocol with a pre-pulse from -100 to 10mV (100 ms duration) and a test pulse at 10 mV (500 ms duration) followed by a repolarizing step at -35 mV. Current density was calculated dividing the current amplitude (pA) by the cell capacitance (pF) for each cell. Steady-state activation and availability curves were fitted with a Boltzmann function: $y = 1/(1 + \exp((V - V_{1/2})/k))$, where y is the relative current, V is the membrane potential, $V_{1/2}$ is the half-maximal voltage, and k is the slope factor.

NaV1.4 sodium channel currents

A 0.5 µg of NaV1.4 plasmid was transiently transfected in HEK293A cells using jetPRIME reagent (PolyPlus transfection, Euroclone) according to the manufacturer's instructions. NaV1.4 sodium currents were measured in whole cell configuration with manual patch-clamp. The intracellular and extracellular solutions, and protocols were the same described for the NaV1.5 current with the exception of the extracellular NaCl concentration that was 60 mM, and of the protocol used to investigate the steady state inactivation, which had the prepulse duration of 100 msec, being the NaV1.4 a skeletal muscle channel.

NaV 1.5 Epitope Mapping

The amino acids sequence of NaV1.5 extracelular loops 1-6 (1(263-368), 2(861-897), 3(1349-1414), 4(1598-1634), 5(1670-1707) and 6(1711-1754) were elongated with neutral GSGSGSG linkers at the N- and C-termini to avoid truncated peptides. The elongated sequences were converted into 15 amino acid peptides with a peptide-peptide overlap of 14 amino acids. The total number of peptides was 327 printed in duplicate (654 peptide spots), and 40 spots of HA control (YPYDVPDYAG) and 38 spots of Polio control (KEVPALTAVETGAT) were included on the array. The custom peptide microarray was pre-stained with the secondary antibody goat anti-human IgG (Fc) DyLight680 (0.1 µg/ml) for 45 min in incubation buffer at RT

to investigate background interactions that could interfere with the main assays. Plasma from BrS patients was applied diluted 1:150, for 16 h at 4°C and orbital shaking at 140 rpm was followed by staining with the secondary antibody. The additional HA peptides framing the peptide microarrays were simultaneously stained with mouse monoclonal anti-HA (12CA5) DyLight800 (0.2 µg/ml) for 45 min at RT. Read-out was performed with an Innopsys InnoScan 710-IR Microarray Scanner. Microarray image analysis was done with PepSlide® Analyzer a software algorithm breaks down fluorescence intensities of each spot into raw, foreground and background signals, and calculates averaged median foreground intensities. Based on averaged median foreground intensities, intensity maps were generated and interactions in the peptide maps highlighted by an intensity color code with red for high and white for low spot intensities.

Transcriptomics analysis

RNA was extracted from 2 ml of fresh blood with Maxwell® RSC simply RNA Blood Kit according to the manufacturer's instructions, measured and stored at -80°C. RNA-seq sequencing analysis of Peripheral Blood mononuclear Cells (PBMCs) from 120 subjects (BrS: 60 subjects, 39 males and 21 females; controls: 60 subjects, 31 males and 29 females). RNA quality was confirmed using Tapestation (Agilent), and only RNAs with an RIN (RNA integrity number) >7 were included in the analysis. To generate RNA libraries, the TruSeq stranded mRNA protocol was performed, starting from a total RNA of 300 nanograms. The RNA libraries were then sequenced on an Illumina NextSeq2000 sequencer, generating 50 M paired-end reads of 75 nucleotides in length for each run. The transcriptomics sequencing data were processed using the Illumina DRAGEN platform (version 3.8.4) with the Illumina DRAGEN RNA pipeline, using the Ensembl 98 Human Genome build (GRCh38.p13) as the reference genome. Differential expression analysis was performed using DESeq2, focusing on protein-coding genes with detectable expression (i.e., gene read counts > 0) in at least 5% of the samples, yielding 14,480 protein-coding genes. The design formula included factors for age, gender, batch analysis, and condition. The complete design formula was: ~ batch + age + gender + condition. Gene Set Enrichment Analysis (GSEA) was performed using WebGestalt (WEB-based Gene SeT AnaLysis Toolkit, http://www.webgestalt.org/)[5].

Z-score-based standardized IFN score calculation

VST-normalized counts were generated using the design formula: ~ batch + age + gender + condition and z-scores for 8 genes closely related to the IFN pathway (*RSAD*, *OAS2*, *ISG15*, *IFIT1*, *IFIT2*, *IFIT3*, *IFITM1*, *IFITM3*) were calculated using the mean and standard deviation of the entire study cohort using the following equation for each gene:

$$Z - score\ for\ each\ gene = \frac{\text{gene count} - \text{mean (gene expression)}}{\text{standard deviation (gene expression)}}$$

Then, the IFN score was calculated by summing the 8 z-scores for each sample. The calculation for each z-score is relative to the mean and standard deviation of the entire population, therefore, each z-score and the summary z-score can become negative if gene expression is below the mean of the population[6].

Plasma IFN-γ levels measurement

Plasma samples from both BrS patients and healthy controls were assessed for the presence of IFN-γ using a High Sensitivity ELISA kit (Thermo Fisher). The assay procedures adhered to the manufacturer's instructions. In summary, plasma was appropriately diluted in sample dilutions, biotin-conjugated, and then incubated for 2 hours at room temperature on a microplate shaker. Following multiple washes, streptavidin-HRP was introduced and incubated for 1 hour at room temperature. Subsequently, Amplification Solution was added to the wells after additional washes and incubated for 15 minutes at room temperature before monitoring color development by pipetting Substrate solution. Finally, the absorbance of each microwell was measured using a spectrophotometer at 450 nm.

Supplementary Results

NaV1.5 channel mapping

Mapping the epitopes of the NaV1.5 protein revealed the identification of six regions predominantly targeted by autoantibodies found in the plasma of BrS patients.:

Peptide 1a: DI-S5-S6 (263-368) SVEADGLVWESLDLYLSDPENYLLKNGTS

Peptide 1b: DI-S5-S6 (263-368) CLKAGENPDHGYTSFDSFAWAFLAL

Peptide 3a: DIII S5-S6 (1349-1414) DSDSGLLPRWHMMDFFHAFLII

Peptide 3b: DIII S5-S6 (1349-1414) VNNKSQCESLNLTGEYWTKVK)

Peptide 4a: DIV S3-S4 (1598-1634) GSGVILSIVGTVLSDIIQKYFFSPT

Peptide 5a:1DIV S5-S6 (1670-1707) MANFAYVKWEAGIDDMFNFQTFANSMLCLF

Peptide 6a: (1711-1754) GWDGLLSPILNTGPPYCDPTLPNSNGSRGD

Effect of NaV1.5 Autoantibodies on NaV1.5 Proteins In-Vitro

The NaV1.5 content on the cell membrane of HEK293A cells overexpressing the channel was determined after a 1 h incubation with BrS patient or control plasma. This assessment was performed using protein biotinylation, as shown in Supplementary Fig. 11A. Western blot analysis showed a significant decrease in membrane-bound (biotinylated) NaV1.5 protein levels after incubation with BrS patient plasma compared to control plasma (Supplementary Fig. 11B-C), demonstrating autoantibody-induced internalization of the channel (Fig. 11D).

The effect of BrS plasma on NaV1.5 current was channel-specific and dose-dependent

The three selected BrS plasmas induced a reduction in current density ranging between 24 and 43% when incubated at a concentration of 5%. At 0.5% plasma concentration, the sodium current was greatly reduced or abolished (Supplementary Fig. 7). The observed effect was specific for the sodium current, as demonstrated by the results obtained in hiPSC-CMs. This model, more complex than heterologous systems of expression, allowed the simultaneous study of the major cardiac inward currents I_{Na} (as TTX-sensitive current) and I_{CaL} (as nifedipine-sensitive current). Results showed that, after 1-h incubation with BrS plasma, I_{Na} density was significantly decreased by approximately 50% compared to untreated cardiomyocytes (Supplementary Fig. 8 and Supplementary Table 6. On the other hand, I_{CaL} density was not affected by the incubation (Supplementary

Fig. 9 and Supplementary Table 7). Furthermore, three different patient plasmas were tested on HEK293A cells overexpressing the Cav3.2 T-type calcium channel, which current was not detected in hiPSC-CMs but plays a significant role in pacemaker tissues and has recently been found in the membrane of mice ventricular myocytes[7]. As a reference, the same plasmas were tested on stable HEK293A cells overexpressing NaV1.5 (a kind gift from Prof. H. Abriel, University of Bern). Automated patch-clamp measurements confirmed an average reduction of 23% in NaV1.5 current density, while no detectable effect was observed on Cav3.2 current density (Supplementary Fig. 9 and Supplementary Table 7). Additionally, affinity/binding experiments identified regions of interest representing a major binding site for BrS-related autoantibodies. Through sequence alignment, a high level of homology with a similar region in the skeletal muscle NaV1.4 sodium channel was found. Thus, three BrS plasmas were tested on HEK293A cells transiently transfected with either NaV1.5 or NaV1.4. A significant reduction in current density was observed for both channels (-43% and -57% for NaV1.5 and NaV1.4, respectively; Supplementary Fig. 10 and Supplementary Table 8). These results clearly suggest the specificity of the effect of BrS patient plasma on sodium current.

Influence of Age and Sex on Test Performance

The correlation between the presence of antibodies and age was tested using the Chi-Square test, which indicated no significant correlation (Chi-Square = 0.04, p-value = 0.83). This finding was further supported by Cramér's V test, showing a very low association value (0.04). Additionally, logistic regression analysis also demonstrated no significant correlation between antibody presence and age (coefficient = 0.01, p-value = 0.38).

a

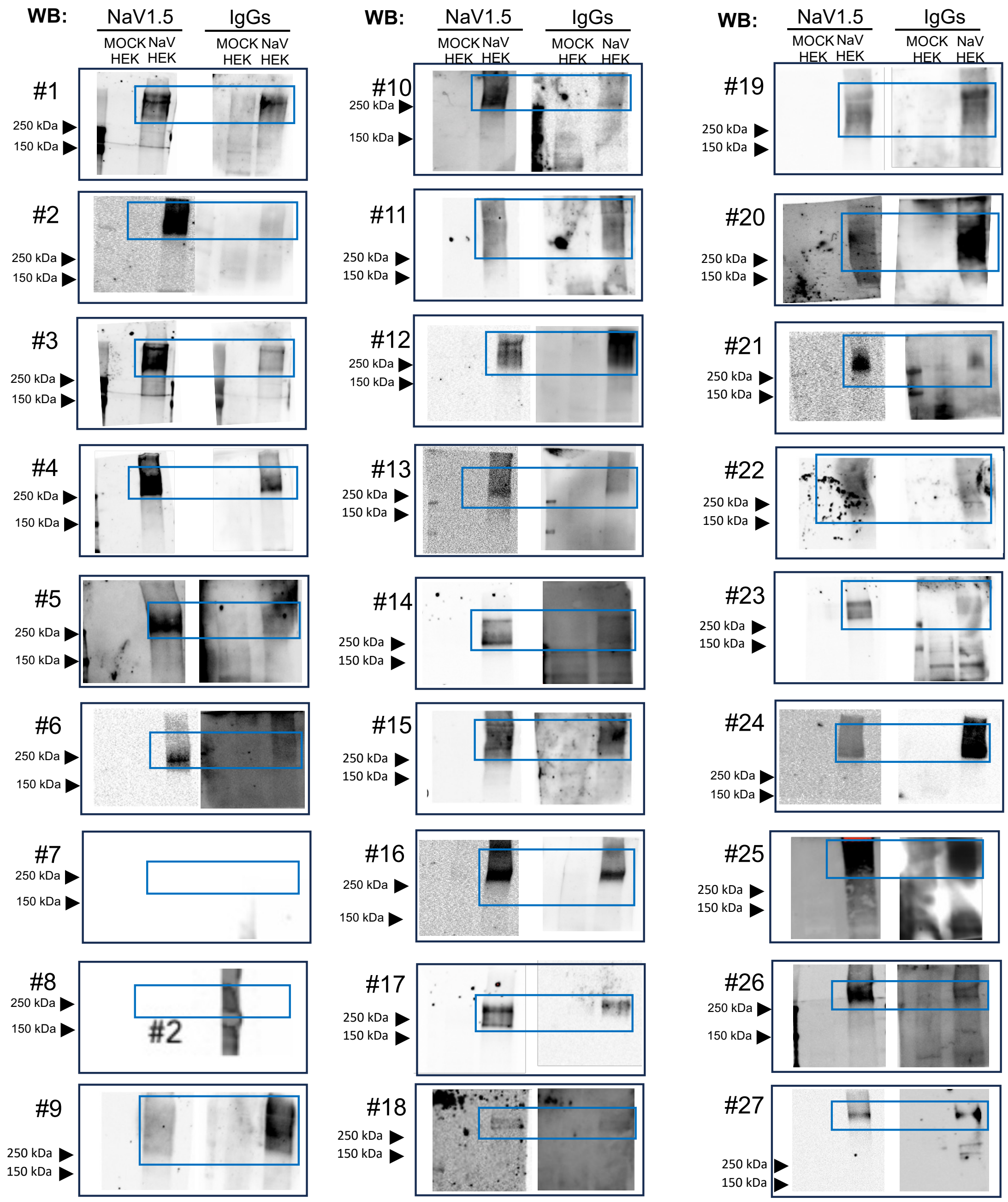
WB:
NaV1.5
IgGs
MOCK HEK
NaV HEK
#1
#2
#3
#4
#5
#6
#7
#8
#9
#10
#11
#12
#13
#14
#15
#16
#17
#18
#19
#20
#21
#22
#23
#24
#25
#26
#27
250 kDa
150 kDa

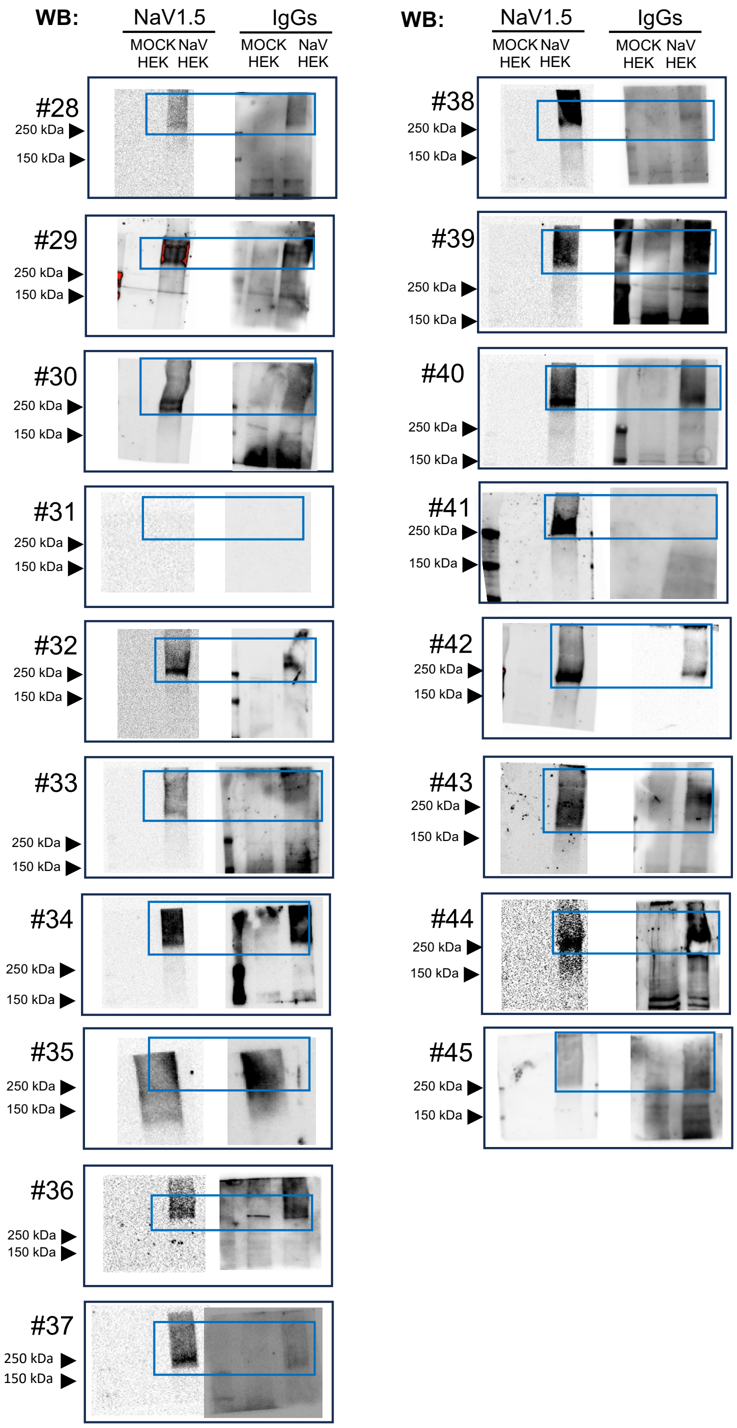
WB:
NaV1.5
IgGs
MOCK HEK
NaV HEK
MOCK HEK
NaV HEK
#28
#29
#30
#31
#32
#33
#34
#35
#36
#37
#38
#39
#40
#41
#42
#43
#44
#45
250 kDa
150 kDa

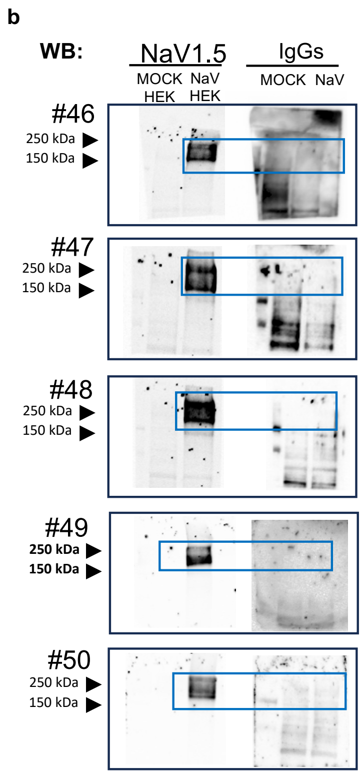
b
WB:
NaV1.5
IgGs
MOCK HEK
NaV HEK
MOCK
NaV
#46
250 kDa
150 kDa
#47
250 kDa
150 kDa
#48
250 kDa
150 kDa
#49
250 kDa
150 kDa
#50
250 kDa
150 kDa

c

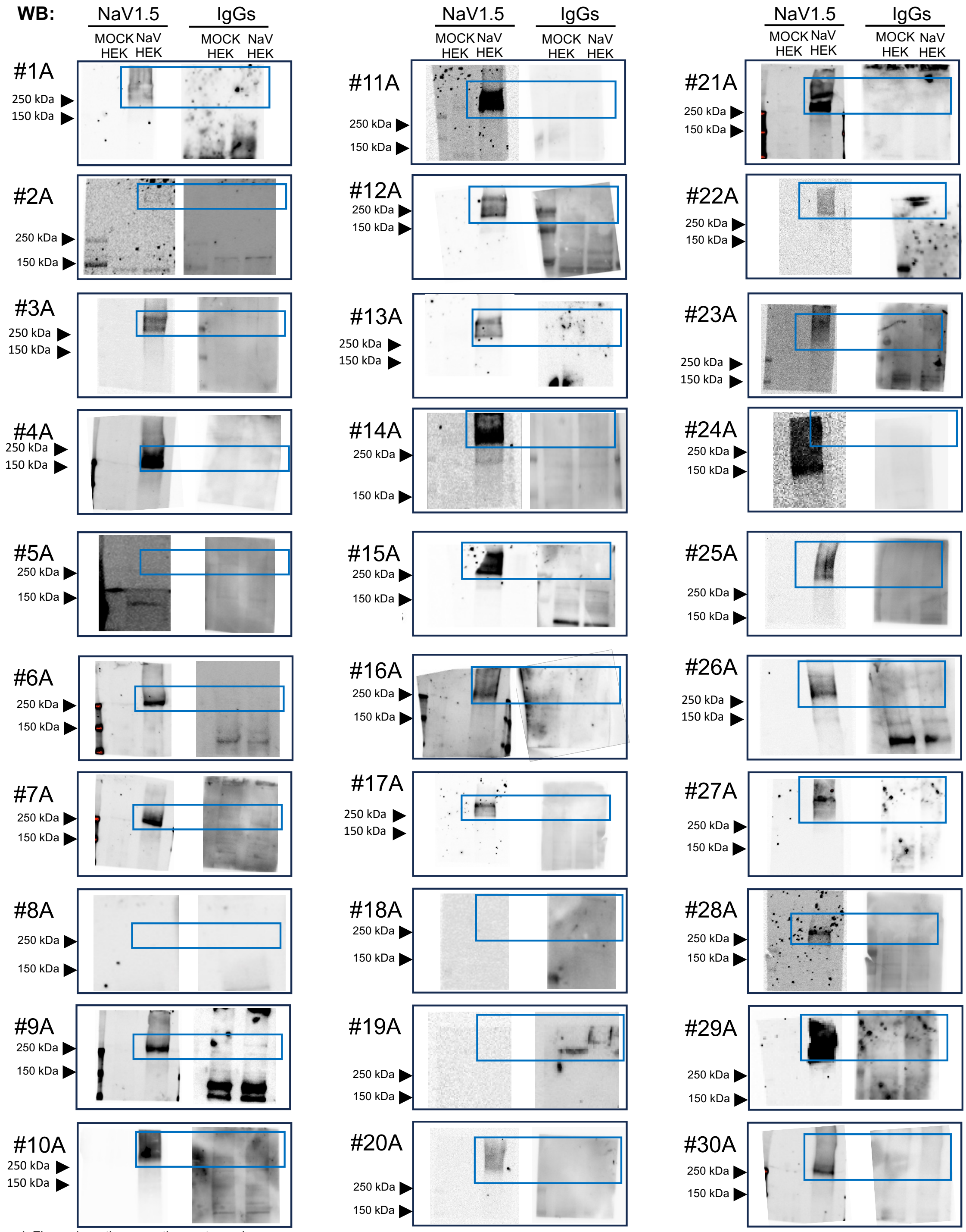

(Suppl. Figure 1 continues on the next page)

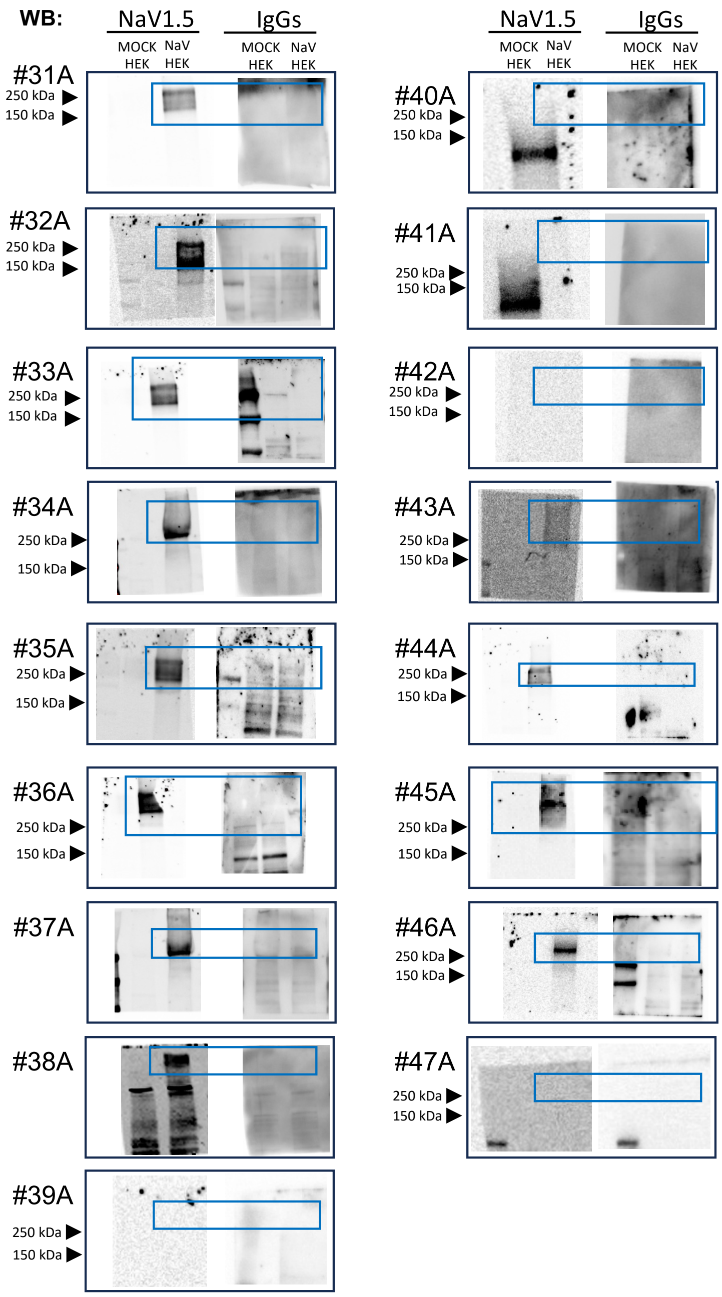
WB:
NaV1.5
IgGs
MOCK HEK
NaV HEK
MOCK HEK
NaV HEK
NaV1.5
IgGs
MOCK HEK
NaV HEK
MOCK HEK
NaV HEK
#31A
250 kDa
150 kDa
#32A
250 kDa
150 kDa
#33A
250 kDa
150 kDa
#34A
250 kDa
150 kDa
#35A
250 kDa
150 kDa
#36A
250 kDa
150 kDa
#37A
#38A
#39A
250 kDa
150 kDa
#40A
250 kDa
150 kDa
#41A
250 kDa
150 kDa
#42A
250 kDa
150 kDa
#43A
250 kDa
150 kDa
#44A
250 kDa
150 kDa
#45A
250 kDa
150 kDa
#46A
250 kDa
150 kDa
#47A
250 kDa
150 kDa

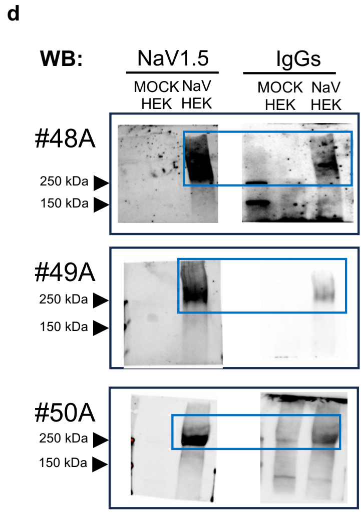

Supplementary Fig. 1. Western Blot analysis to evaluate the presence of the NaV1.5 autoantibodies in the study cohort. (A) BrS patients showing the presence of anti-NaV1.5 autoantibodies; (**B)** BrS patients negative for the presence of anti-NaV1.5 autoantibodies; **(C)** Healthy controls negative for the presence of anti-NaV1.5 autoantibodies; **(D)** Healthy controls positive for the presence of anti-NaV1.5 autoantibodies.

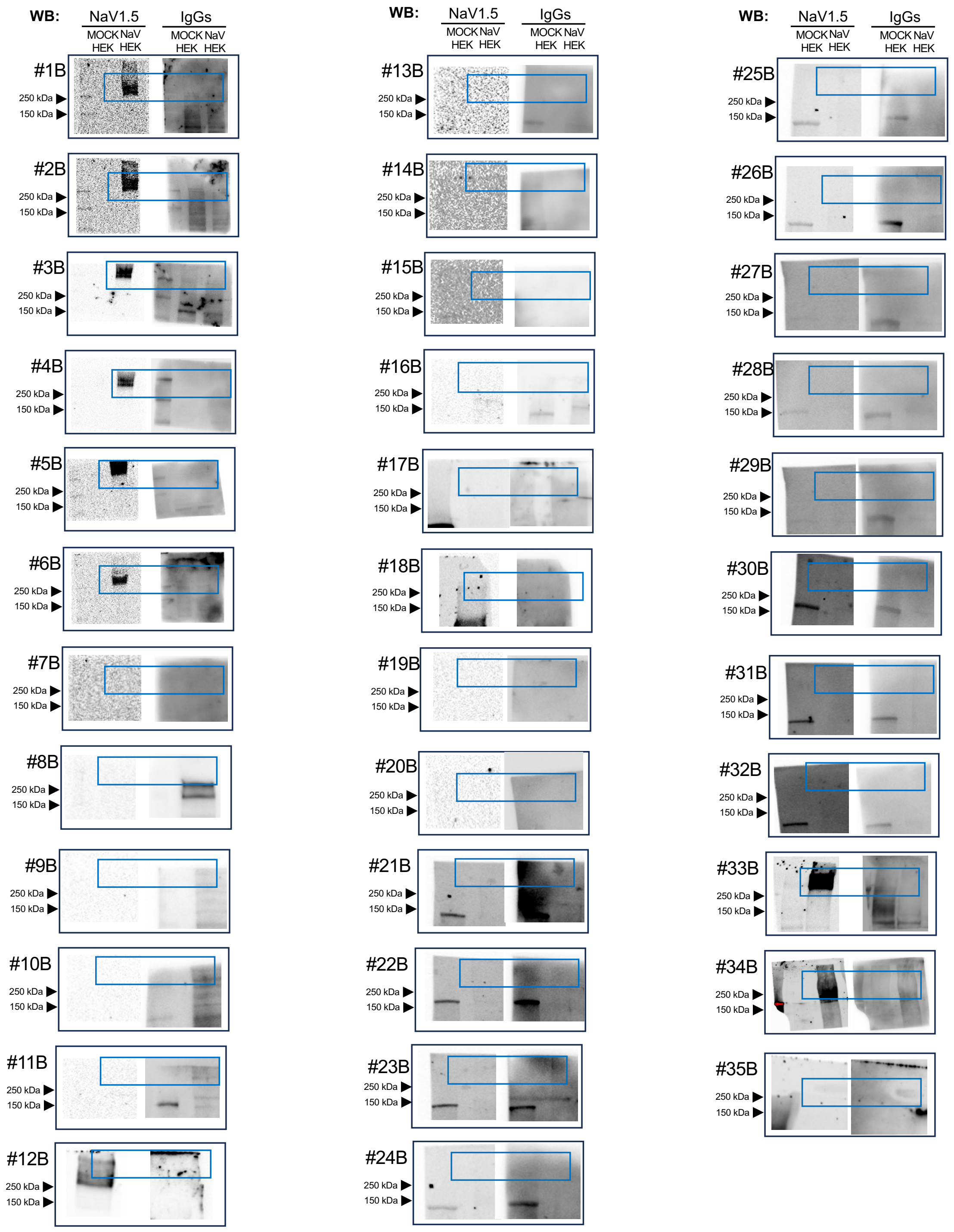

Supplementary Fig. 2. Western Blot analysis to evaluate the presence of the NaV1.5 autoantibodies in the study cohort of 35 with other cardiac diseases, including channelopathies such as long QT syndrome, heart failure, and cardiomyopathies.

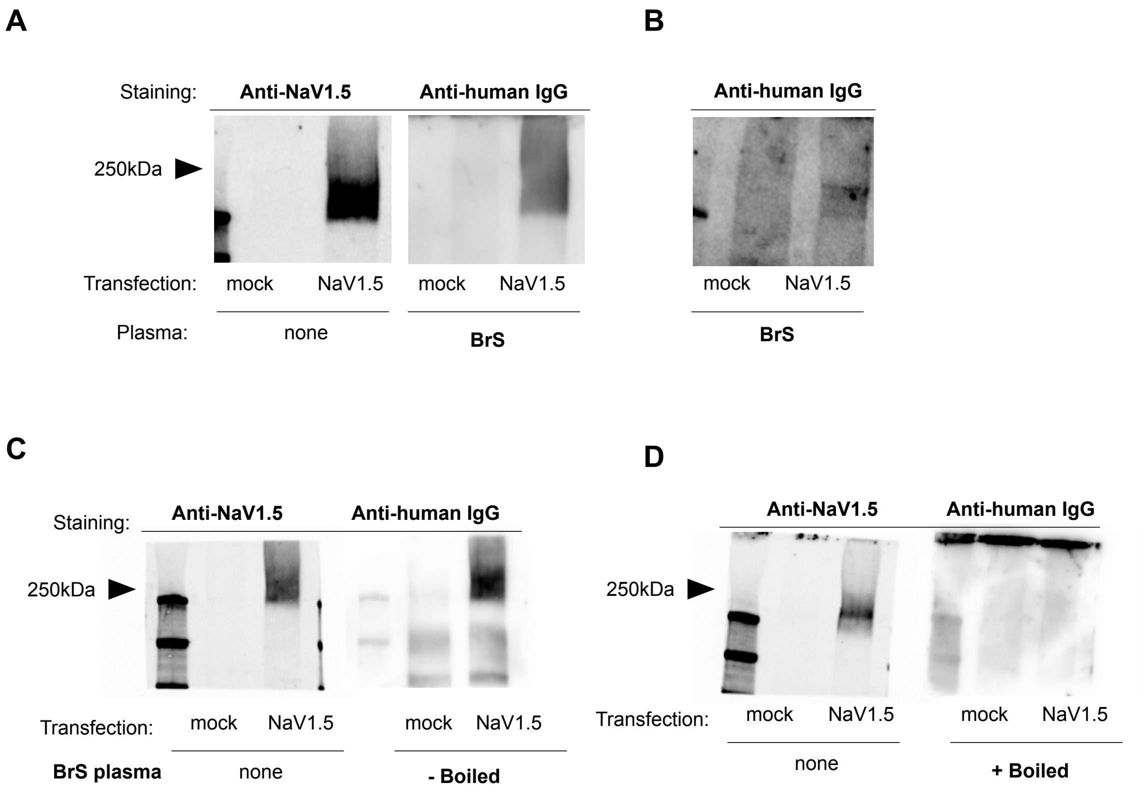

Supplementary Figure 3. Binding Specificity of BrS Patient Plasma IgGs to NaV1.5 Protein. (**A**) Western blot analysis using an anti-NaV1.5 antibody demonstrates the presence of NaV1.5 protein in cell lysates from NaV1.5-transfected HEK293A cells, but not in mock-transfected cells. Incubation with BrS patient plasma, followed by staining with an anti-human IgG antibody, illustrates specific binding of autoantibodies from BrS patients to NaV1.5; (**B**) Direct exposure of NaV1.5-transfected cell lysates to BrS patient plasma, without pre-incubation with anti-NaV1.5, followed by an anti-human IgG antibody reveals specific autoantibody binding; (**C**) Lysates pre-stained with anti-NaV1.5 antibody and subsequently treated with BrS patient plasma and an anti-human IgG antibody confirm the specific interaction of BrS patient autoantibodies with NaV1.5; (**D**) Lysates incubated with BrS patient plasma that was boiled prior to exposure demonstrate loss of specific anti-NaV1.5 IgG binding to the NaV1.5 protein, indicating that heat denaturation of IgGs abolishes their binding capability.

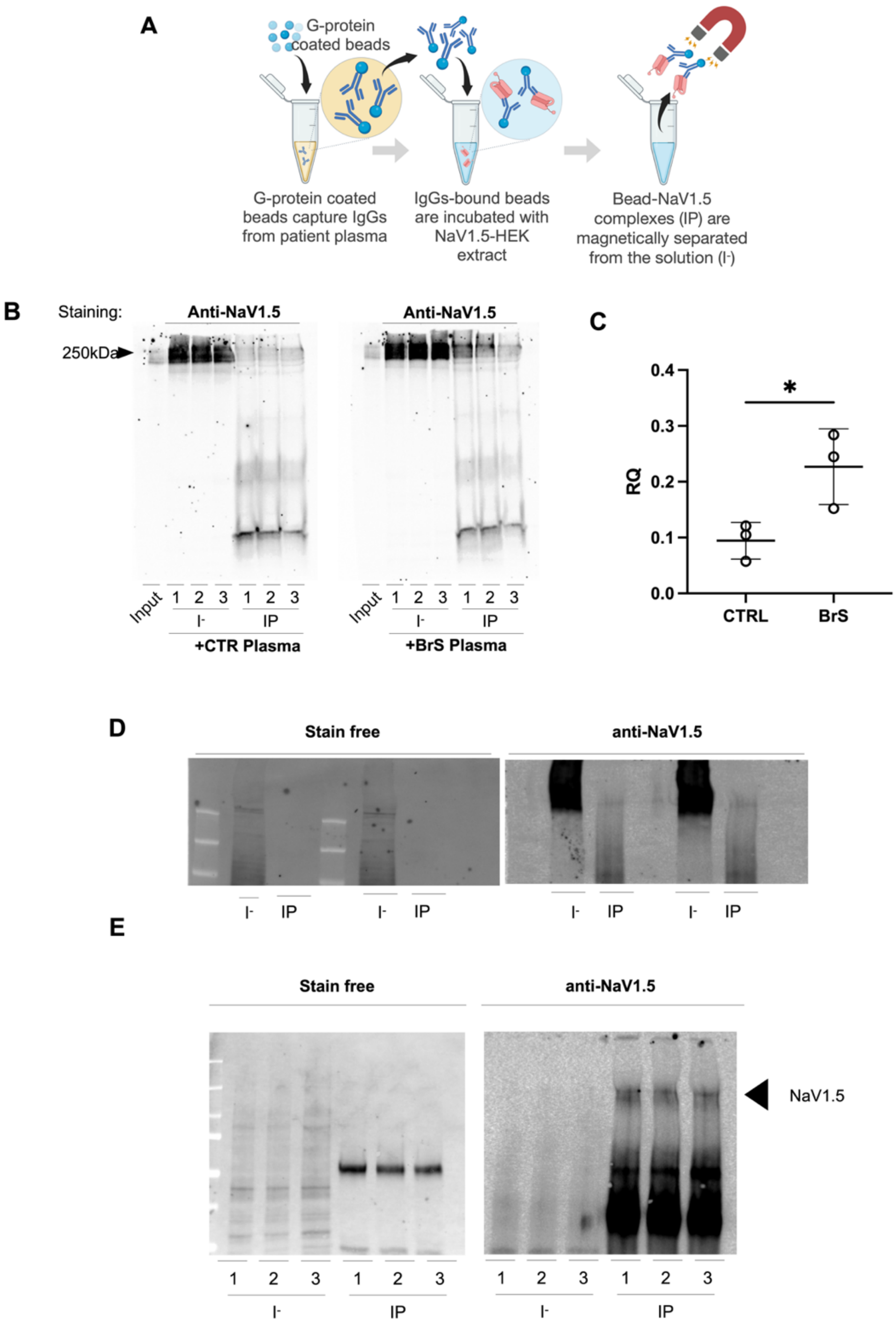

Supplementary Fig. 4. Specific Binding of BrS Patient Autoantibodies to NaV1.5 Channel Protein. (**A**) Schematic of the immunoprecipitation (IP) methodology employed to isolate and analyze the interaction between NaV1.5 channel protein and IgGs derived from patient plasma. Protein G-coated beads are used to capture IgGs, which are then incubated with cell lysates from

HEK293A cells overexpressing NaV1.5 to form IgG-NaV1.5 complexes. These complexes are magnetically separated and subsequently assessed via western blot for specific NaV1.5 capture; (**B**) Western blot post-IP demonstrates the presence of NaV1.5 in the immunoprecipitated samples, indicated by the anti-NaV1.5 antibody. Comparative analysis reveals specific affinity of IgGs from BrS patient plasma for NaV1.5, as opposed to the lack of binding with control plasma; (**C**) Graphic quantification of NaV1.5 protein detected in the immunoprecipitated samples normalized on total protein run; (**D**) Validation of the IP process showing the western blot of the immunoprecipitated (IP) and immunodepleted (I-) fractions after incubation with protein lysate from HEK293A cells overexpressing NaV1.5. The absence of non-specific binding of NaV1.5 to empty beads is confirmed, as well as the presence of NaV1.5 in the IP fraction using anti-NaV1.5 antibody staining; (**E**) Immunoprecipitation assay utilizing mouse cardiac ventricular protein extract and magnetic beads coated with IgGs from BrS patient plasma. Western blot analysis of IP and I- fractions, stained with an anti-NaV1.5 antibody, confirms specific binding of BrS autoantibodies to NaV1.5 in cardiac tissue.

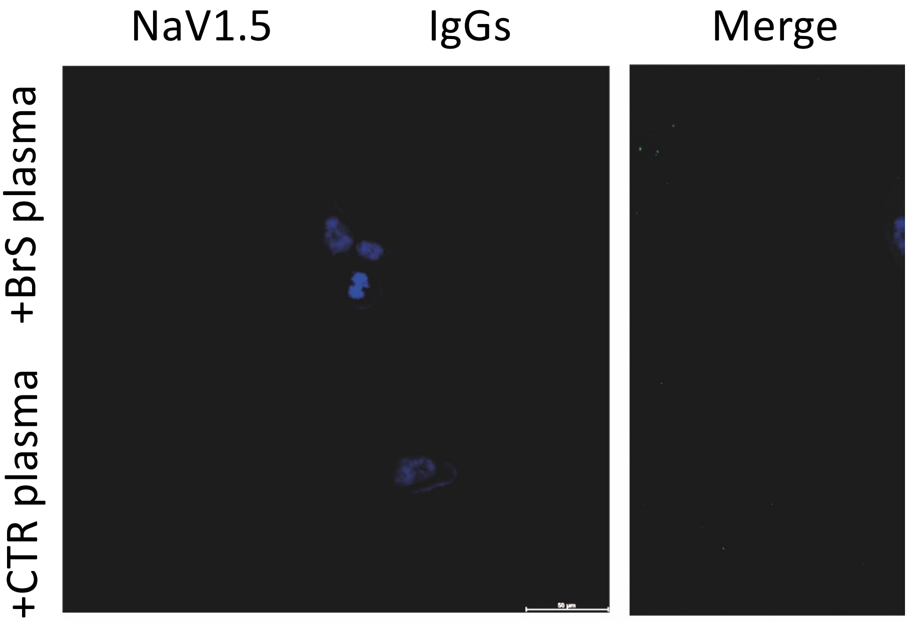

Supplementary Fig.5. Validation of NaV1.5 targeting by plasma IgG in BrS.

Immunofluorescence of NaV1.5 MOCK-HEK293A cells shows no reactivity for NaV1.5 (red) and IgGs (green), after incubation with BrS or CTR plasma.

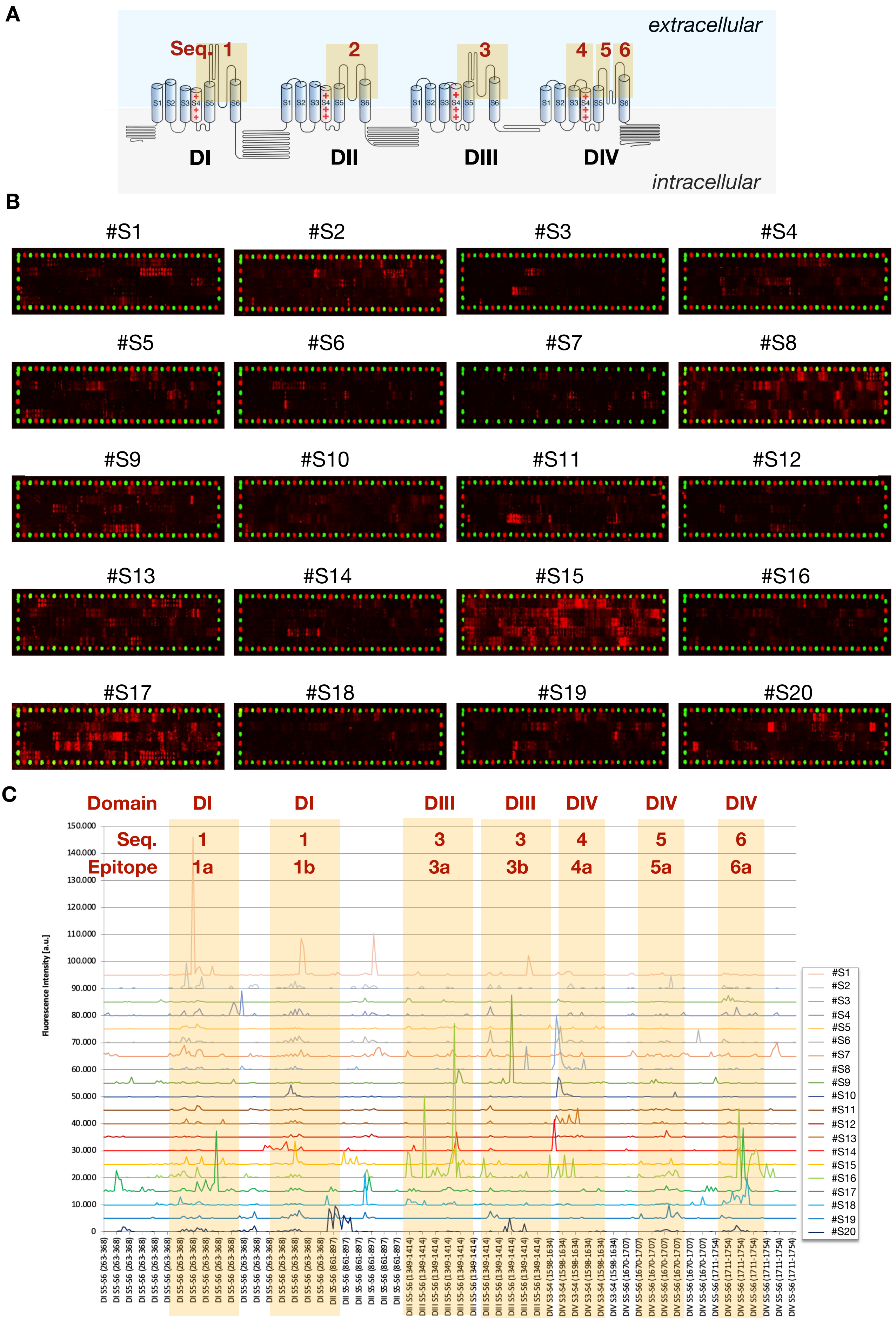
A
extracellular
intracellular
Seq. 1
2
3
4 5 6
S1 S2 S3 S4 S5 S6
DI
DII
DIII
DIV
B
#S1
#S2
#S3
#S4
#S5
#S6
#S7
#S8
#S9
#S10
#S11
#S12
#S13
#S14
#S15
#S16
#S17
#S18
#S19
#S20
C
Domain DI DI DIII DIII DIV DIV DIV
Seq. 1 1 3 3 4 5 6
Epitope 1a 1b 3a 3b 4a 5a 6a
Fluorescence Intensity [a.u.]
150.000
140.000
130.000
120.000
110.000
100.000
90.000
80.000
70.000
60.000
50.000
40.000
30.000
20.000
10.000
0
DI S5-S6 (263-368)
DII S5-S6 (861-897)
DIII S5-S6 (1349-1414)
DIV S3-S4 (1598-1634)
DIV S5-S6 (1670-1707)
DIV S5-S6 (1711-1754)

Supplementary Fig. 6: Autoantibody Epitope Mapping on NaV1.5. (**A**) Diagram of NaV1.5 channel topology highlighting the extracellular loop regions (Seq. 1-6) analyzed in epitope mapping. These loops, located between transmembrane domains, identify different epitopes as the primary interaction sites for circulating autoantibodies in BrS; (**B**) High-density peptide microarray results displaying specific epitopes on NaV1.5 extracellular loops where BrS patient autoantibodies bind. Each peptide segment is represented as a 15-mer sequence for comprehensive coverage. Red fluorescence indicates regions of higher antibody affinity, providing qualitative data on binding interactions; (C) Intensity plot aggregating data from patient plasma samples against NaV1.5 domain segments: DI S5-S6 (263-368), DII S5-S6 (861-897), DIII S5-S6 (1349-1414), DIV S3-S4 (1598-1634), DIV S5-S6 (1670-1707), and DIV S5-S6 (1711-1754). The plots visualize aggregate binding intensities and signal-to-noise ratios, correlating peptide sequences with intensity mapping to identify prominent autoantibody epitopes across patient samples. The diagram highlights consensus epitope regions on NaV1.5 recognized by autoantibodies from the majority of analyzed BrS patients (Epitopes 1a, 1b, 3a, 3b, 4a, 5a, 6a). Full epitope sequences and methodology are detailed in the Supplementary Methods.

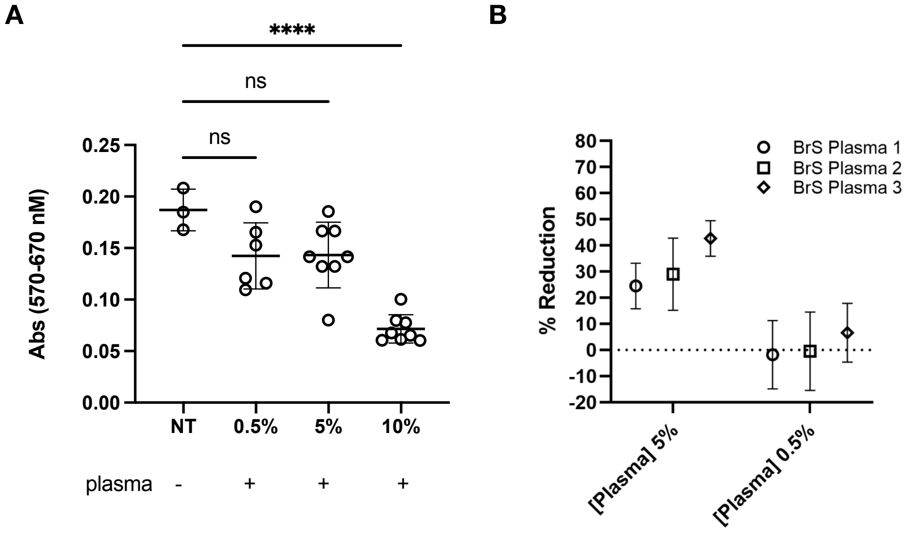

Supplementary Fig. 7 Dose-dependent effect of BrS patient plasma on HEK293A overexpressing NaV1.5. (A) Cell viability post-treatment with varying concentrations of BrS plasma was quantified using the MTT assay. Plasma concentrations of 0.5% and 5% did not affect cell viability, while a 10% concentration resulted in a significant decrease compared to non-treated (NT) controls (****p<0.0001); (**B)** Analysis of current density reduction following exposure to BrS plasma revealed a concentration-dependent effect. The percentage reduction in current density was determined in cells exposed to 5% (left) or 0.5% (right) BrS patient plasma calculated relative to the mean current density observed in untreated cells derived from the same batch and assessed on the same experimental day. For 5% BrS plasma, reductions of 24.5±8.7%, 29.0±13.8%, and 42.7±6.8% were observed (n=11, 9, and 8, respectively), while for 0.5% BrS plasma, reductions of -1.8±13.1%, 0.42±15.0%, and 6.6±11.2% were measured. (n=11, 12, and 13, respectively).

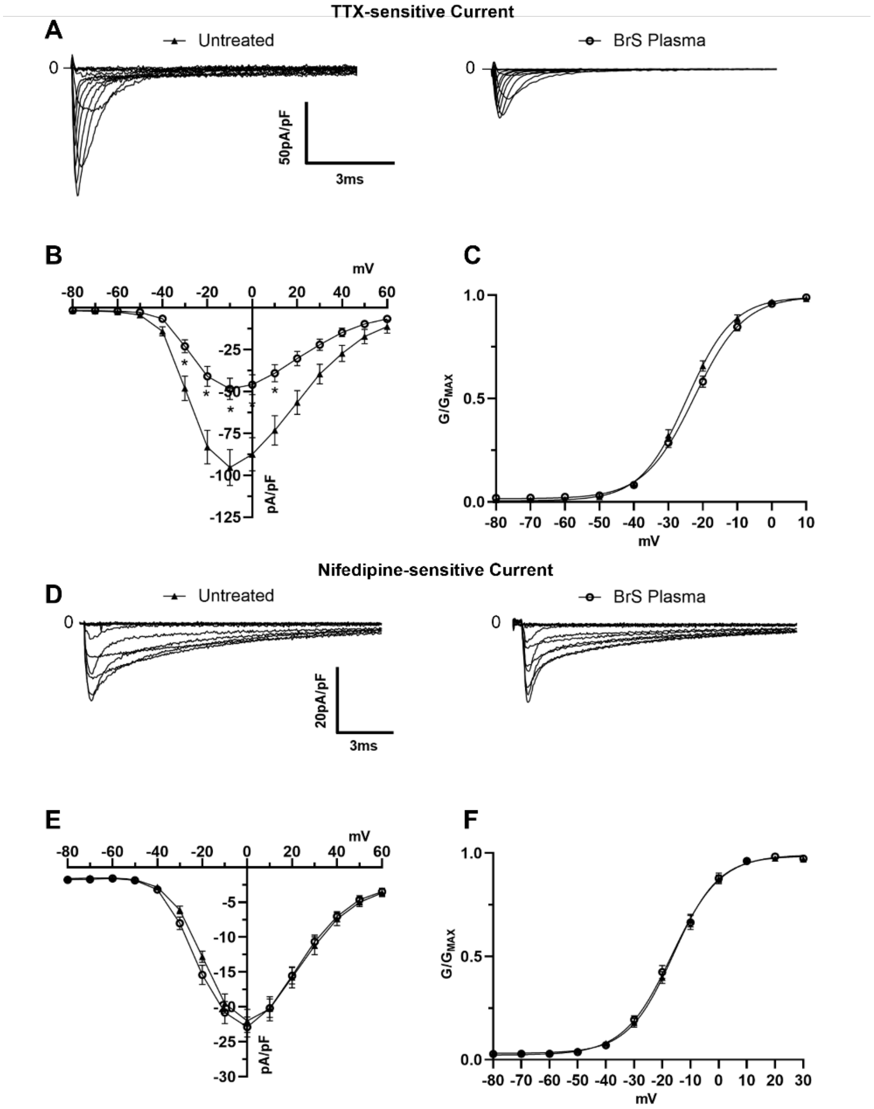

Supplementary Fig.8. Effect of BrS patients' plasma on inward currents in hiPSC-derived cardiomyocytes (hiPSC-CMs). (A) Families of TTX-sensitive sodium current elicited by a voltage steps protocol (holding potential of -80 mV, steps from -80 to 60 mV, duration 50 ms) in untreated (left) or after 1-h incubation with BrS patient plasma (right) showing a ~50% decrease in current density compared to untreated cardiomyocytes. **(B, C)** Mean current-voltage relationship and voltage-dependence of channel activation, respectively, of TTX-sensitive sodium current obtained from untreated cells (black triangles, n=26, N=3 distinct differentiations) or cells incubated with BrS patients' plasma (empty circles, n=33, N=3 plasma) (*p<0.5). (**D**) Families of nifedipine-sensitive calcium current in hiPSC-CMs untreated (left) or incubated with BrS patients' plasma (right), showing that the current density is unaffected by the plasma incubation. (**E, F**) I-V curve and voltage-dependence of activation, respectively, of nifedipine-sensitive calcium current in

untreated cells (black triangles, n=22, N=3 distinct differentiations) and cells incubated with BrS patient plasma (n=30, N=3 BrS plasma). These results strongly suggest for the specificity of the effect of BrS plasma on the sodium current. For all the values, see Supplementary Table 6.

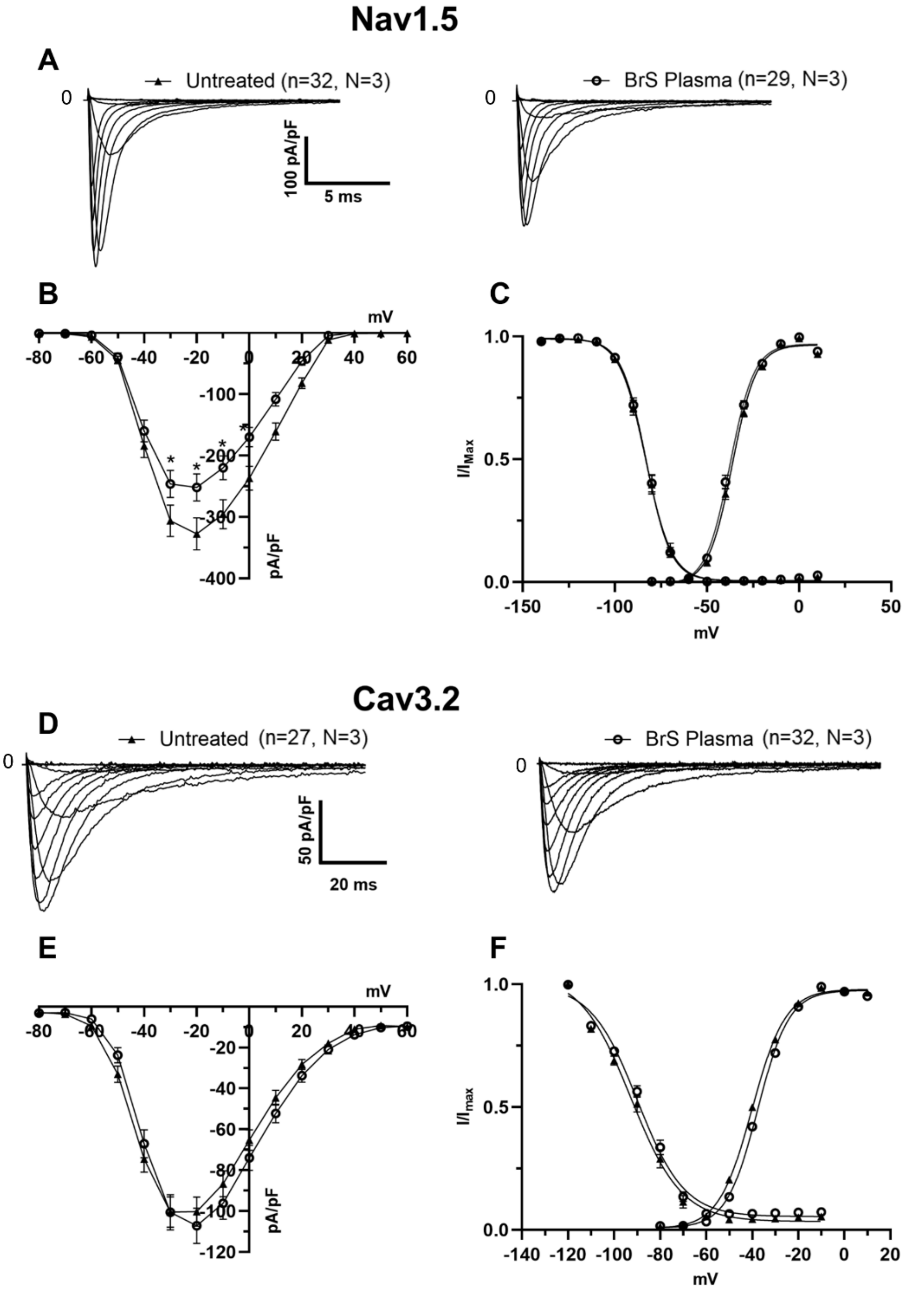

Supplementary Fig.9. Functional effects of the same 3 BrS plasma on HEK293A cells overexpressing NaV1.5 (A-C) or Cav3.2 T-type calcium current (D-F). (**A, D**). Families of NaV1.5 and Cav3.2 T-type calcium current, respectively, elicited by a voltage steps protocol applied through automated patch-clamp, in untreated (left, black triangles) or in cells incubated for 1-h with 5% BrS plasma (right, empty circles). (**B, E**) Mean of current-voltage relationship of NaV1.5 sodium and Cav3.2 T-type calcium current. The results show a significantly average of 23% reduction in NaV1.5, while no detectable effect was observed on Cav3.2 current density. (*p<0.5; values in Supplementary Table 7). (**C, F**) Voltage-dependence activation and inactivation of

NaV1.5 and Cav3.2 T-type calcium current, respectively ($V_{1/2}$ and k value in Supplementary Table 7). These results clearly suggest the specificity of the effect of BrS patient plasma on NaV1.5 current.

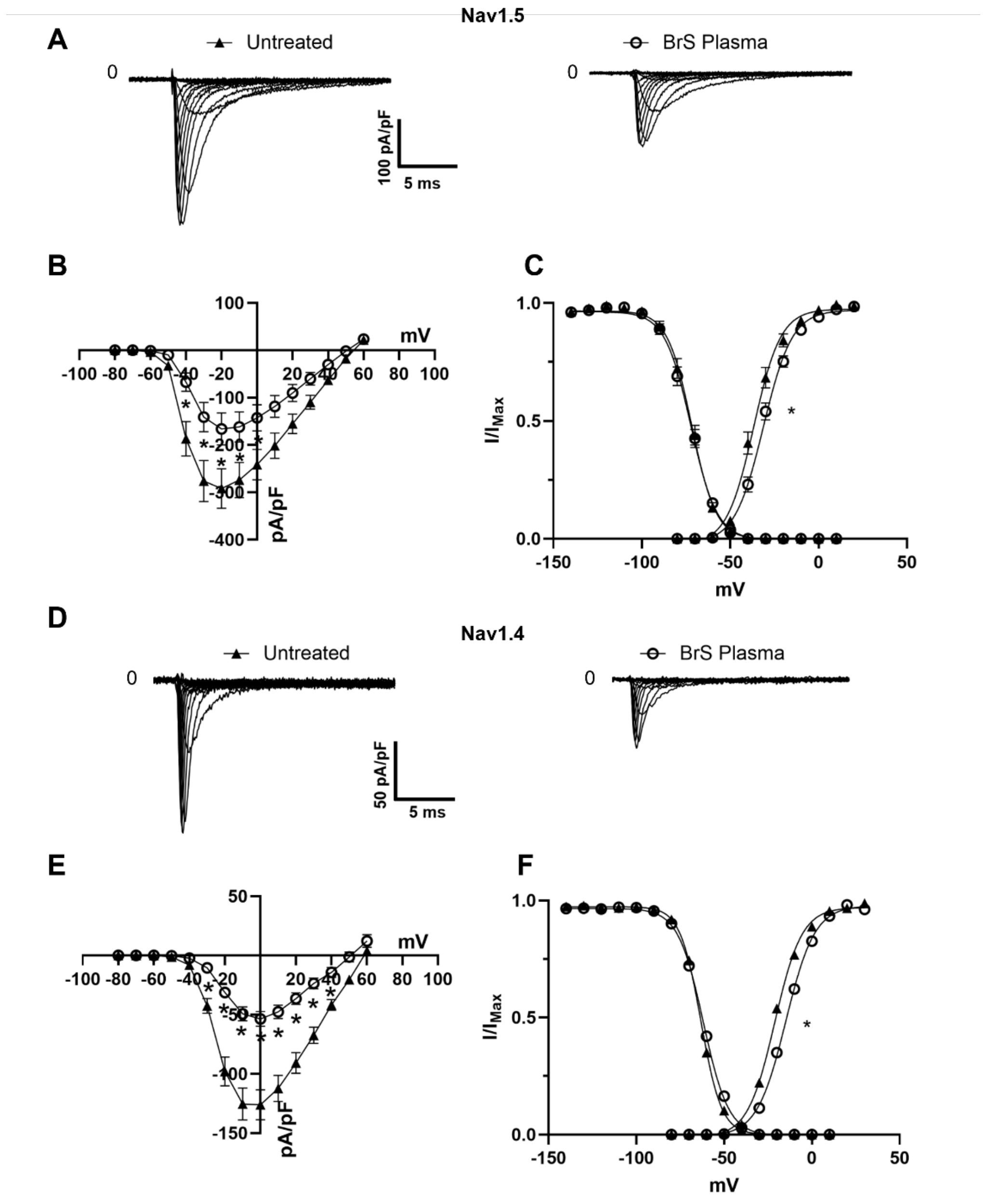

Supplementary Fig.10 Functional effects of 3 different BrS patients' plasma in HEK293A cells transiently overexpressing NaV1.5 or NaV1.4. Since the sequence alignment of the loops identified in NaV1.5 as relevant for autoantibodies binding, we tested the same BrS patients' plasma on HEK293A cell transiently transfected with either NaV1.5 or NaV1.4 (kindly provided by Dr. Serena Pagliarani, IRCCS Fondazione Ca' Granda Ospedale Maggiore Policlinico, Milano) and found similar effects on the two isoforms NaV1.5 and NaV1.4. (**A**) Families of NaV1.5 sodium current recorded in untreated cells (left) or incubated with BrS patient plasma (right); the current density is reduced by ~43%. **(B**) IV-curve relations in untreated cells (black triangles, n=15, N=3) and in cells incubated with BrS patient plasma (empty circles, n=18, N=3)

(*p<0.5). (**C**) Voltage-dependence of NaV1.5 channel activation and inactivation in untreated cells or incubated with BrS patient plasma (*p<0.5). (**D**) Families of NaV1.4 sodium current recorded in untreated cells (left) or incubated with BrS patient plasma (right); the current density is reduced by ~57%. (**E**) I-V curve obtained in untreated cells (black triangles, n=23 N=3) and in cells incubated with BrS patient plasma (empty circles, n=31, N=3) (*p<0.5). (**F**) Voltage-dependence of NaV1.4 channel activation and inactivation (obtained with a typical two-steps protocol in which the pre-pulse duration was 100 ms) in untreated cells or incubated with BrS patient plasma (*p<0.5). The incubation with BrS plasma caused a significant 5 mV rightward shift in the voltage-dependence of activation in both NaV1.5 and NaV1.4. These results indicate that BrS-related anti-NaV1.5 autoantibodies can bind and affect the functionality of other sodium channel isoforms. For all the values, see Supplementary Table 8.

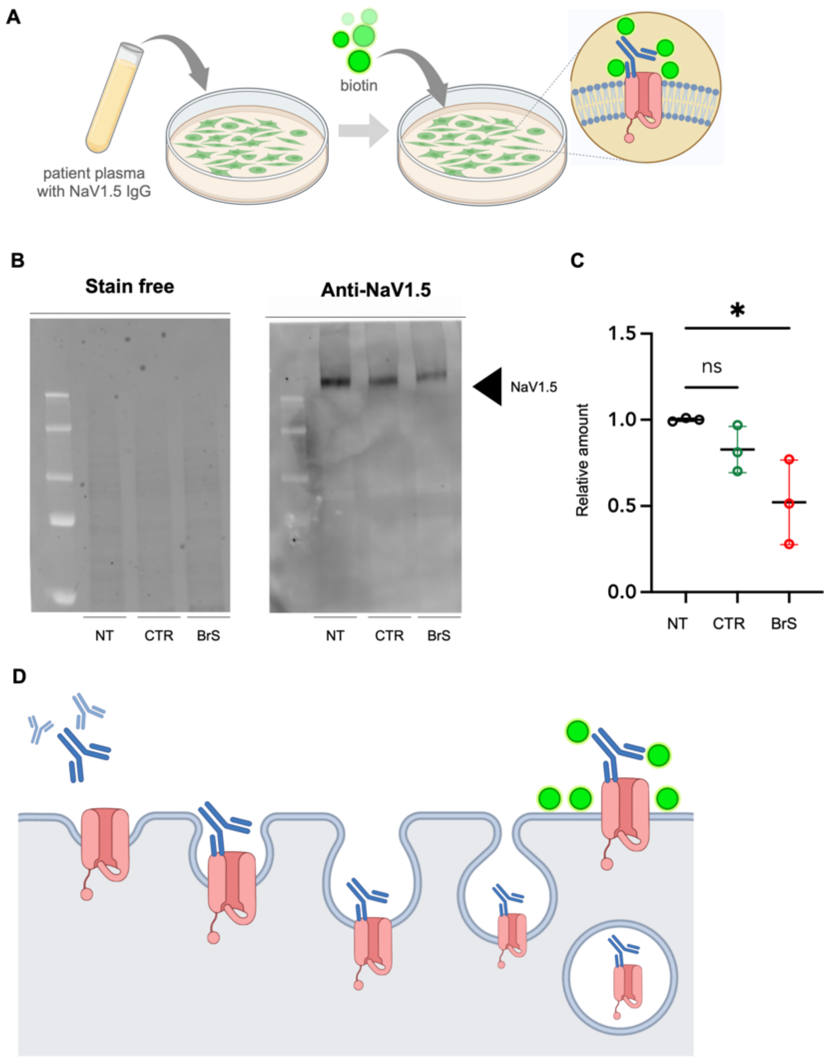

Supplementary Fig. 11. ***Effects of NaV1.5 autoantibodies on NaV1.5 protein in-vitro.*** (**A**) Workflow diagram illustrating the biotinylation assay used to assess NaV1.5 channel internalization; (**B**) western blot detecting biotinylated NaV1.5 channels from untreated (NT), control (CTR), and BrS plasma-treated cells. HEK293A cells over-expressing NaV1.5 were incubated with plasma from BrS patients or controls, followed by biotinylation of cell membrane proteins. Immunoprecipitation with streptavidin-coated beads was performed to quantify membrane-associated NaV1.5. Stain free of total protein for NaV1,5 normalization (left), NaV1.5 staining with a commercial anti-NaV1.5 antibody (right); (**C**) Quantitative analysis of the biotinylated NaV1.5 levels. The graph depicts a significant reduction in the relative amount of membrane-associated NaV1.5 in BrS plasma-treated cells compared to controls, consistent with antibody-induced internalization (*$p<0.05$); (**D**) Conceptual illustration of the proposed mechanism whereby

interaction with autoantibodies from BrS patient plasma leads to partial internalization of the NaV1.5 channel, potentially contributing to the pathophysiology of BrS.

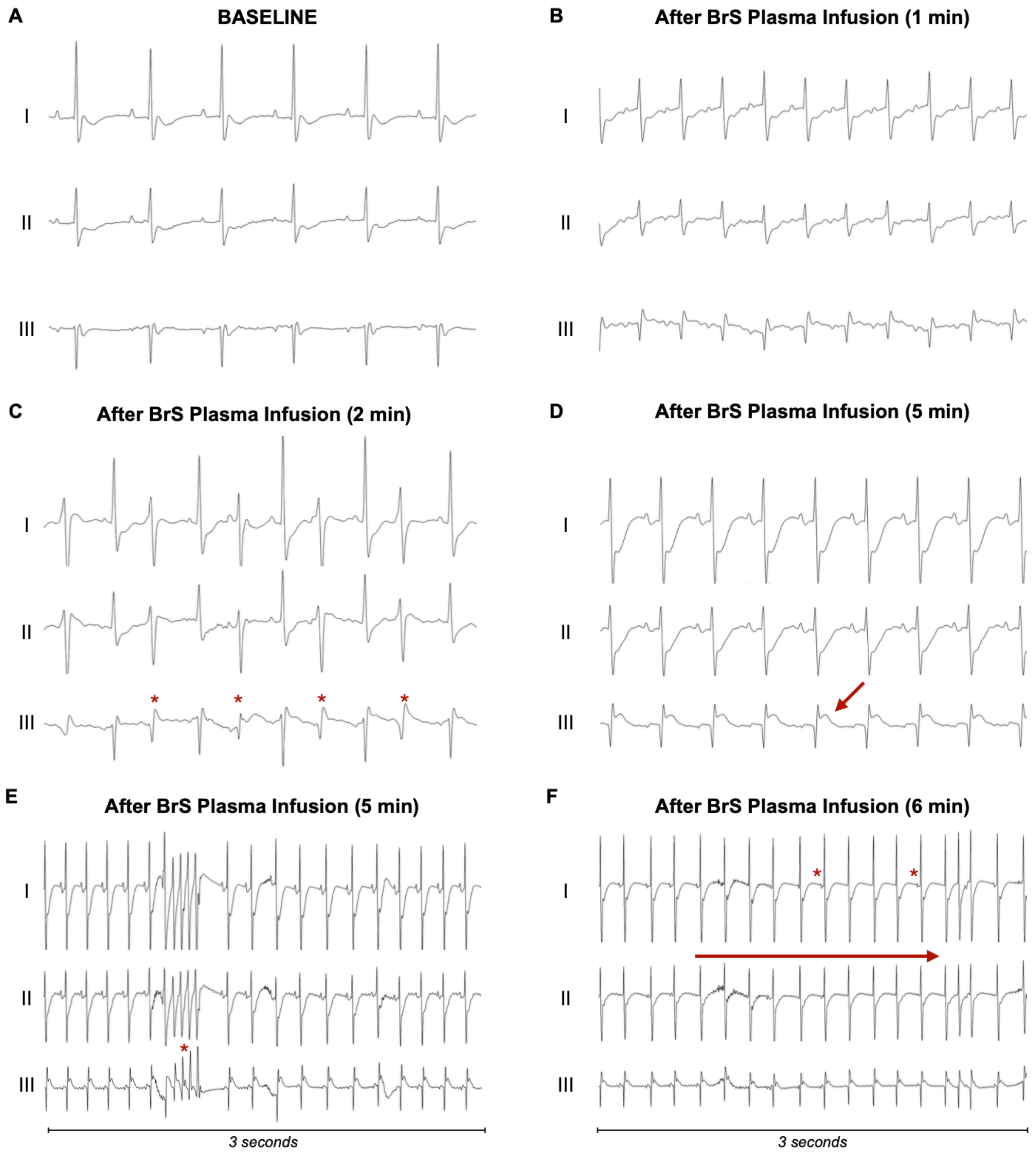

A
BASELINE
B
After BrS Plasma Infusion (1 min)
C
After BrS Plasma Infusion (2 min)
D
After BrS Plasma Infusion (5 min)
E
After BrS Plasma Infusion (5 min)
F
After BrS Plasma Infusion (6 min)
I
II
III
3 seconds
3 seconds

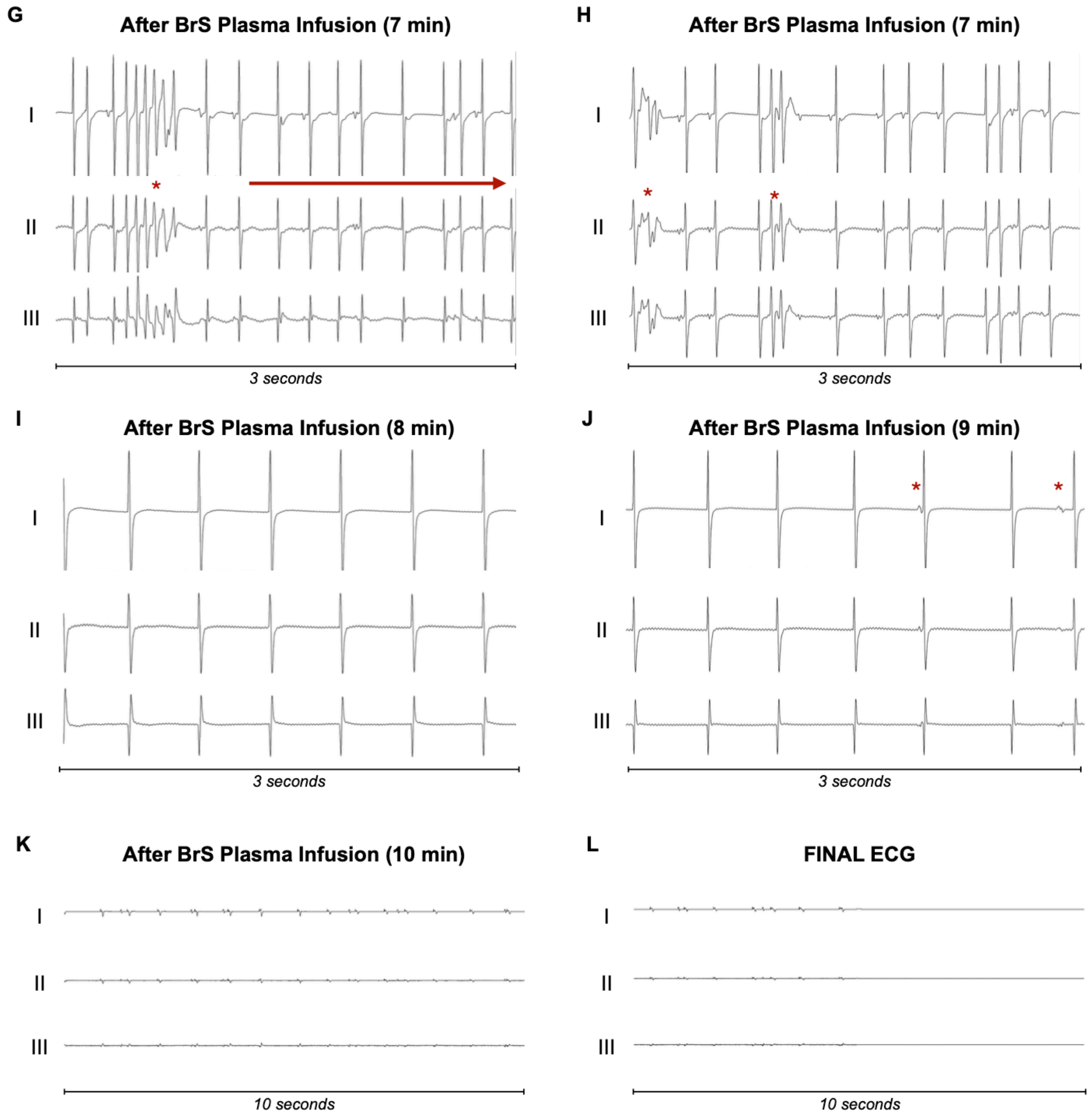

Supplementary Figure 12. Experimental mouse #1 exposed to BrS patient plasma. Sequential electrocardiographic changes in a mouse model following intravenous infusion of plasma from a BrS patient. The panels demonstrate a continuum of arrhythmic manifestations culminating in fatal electromechanical dissociation and death of the mouse following exposure to BrS patient plasma. The ECG recordings are shown in a 1-second time span **(panels A-D),** 3-second time span **(panels E-J)** and 10-second span **(panels K-L),** respectively. (**A**) Baseline ECG with normal sinus rhythm; (**B**) Mild ST segment changes observed during the infusion of plasma (red arrow); (**C**) ST-segment elevation and frequent premature complexes at the end of infusion (red asterisk), indicating a proarrhythmic effect; (**D**) Five minutes post-infusion, significant ST coved-

type elevation is present indicated by the red arrow in lead III with reciprocal ST depression in leads I and II; (**E**) Five minutes post-infusion, the ECG shows Brugada-like ST elevation with non-sustained ventricular arrhythmia indicated by the red asterisk; (**F**) Six minutes post-infusion, coved-type ST-segment elevation persists alongside complete AV block dissociation (red asterisk) and junctional escape rhythm (red arrow) indicative of the severe sodium channel blockade; (**G**) Seven minutes post-infusion, non-sustained polymorphic ventricular tachycardia (red asterisk) emerges with associated AV dissociation (red arrow); (**H**) frequent ventricular couplets (red asterisk); (**I**) Eight minutes after infusion, junctional escape rhythm, with sinus node function suppressed; (**J**) Nine minutes post-infusion, persistent junctional escape rhythm with AV-dissociation (red asterisk); (**K**) Progressive reduction in QRS amplitude, persistent atrioventricular block, and junctional escape rhythm; (**L**) Electromechanical dissociation and asystole lead to the death of the mouse. Same ECG lead configuration as in Figure 4 of the main text.

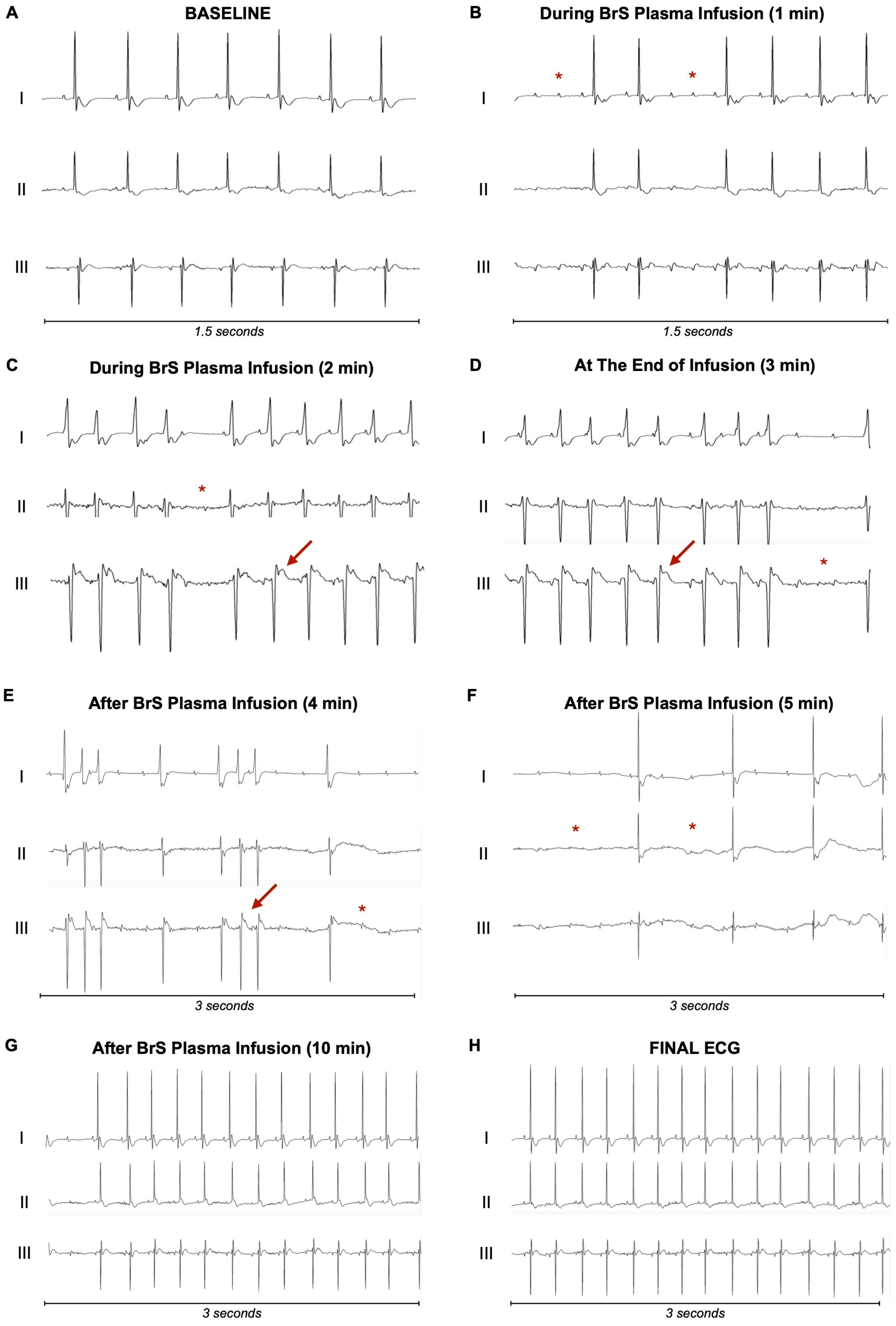
A
BASELINE
B
During BrS Plasma Infusion (1 min)
I
II
III
1.5 seconds
1.5 seconds
C
During BrS Plasma Infusion (2 min)
D
At The End of Infusion (3 min)
E
After BrS Plasma Infusion (4 min)
F
After BrS Plasma Infusion (5 min)
3 seconds
3 seconds
G
After BrS Plasma Infusion (10 min)
H
FINAL ECG
3 seconds
3 seconds

Supplementary Figure 13. Experimental mouse #2 exposed to BrS patient plasma. The panels illustrate a continuum of ECG manifestations culminating in complete AV block, which is a typical reaction due to the severe blockade of the sodium channel, and coved type ST-segment elevation. The ECG recordings are shown in a 1.5-second **(panels A-D)** and 3-second time span **(panels E-H).** (**A**) displays the baseline ECG showing normal sinus rhythm before the plasma infusion; (**B**) indicates the emergence of complete atrioventricular (AV) block during plasma infusion indicated by the red asterisks, characteristic of severe sodium channel blockade effects; (**C**) reveals advanced AV block coupled (red asterisk) with pronounced coved-type ST-segment elevation (red arrow), indicating the proarrhythmic impact of the plasma. Persistent coved-type ST elevation in lead III (red arrow) with reciprocal ST depression in leads I and II and complete AV block (red asterisk), even at the conclusion of plasma infusion is shown in (**D**); (**E-F**) illustrate sustained third-degree AV block (red asterisk) with severe bradycardia, with a partial recovery from coved-type ST elevation (red arrow) observed in (**F**); (**G**) indicates recovery from both complete AV block (red asterisk) and the Brugada-like ECG pattern; (**H**) conveys the final ECG recording ten minutes post-infusion, with a resolution of the arrhythmic changes, ending the experiment with the mouse alive and stable. Same ECG lead configuration as in Figure 4 of the main text.

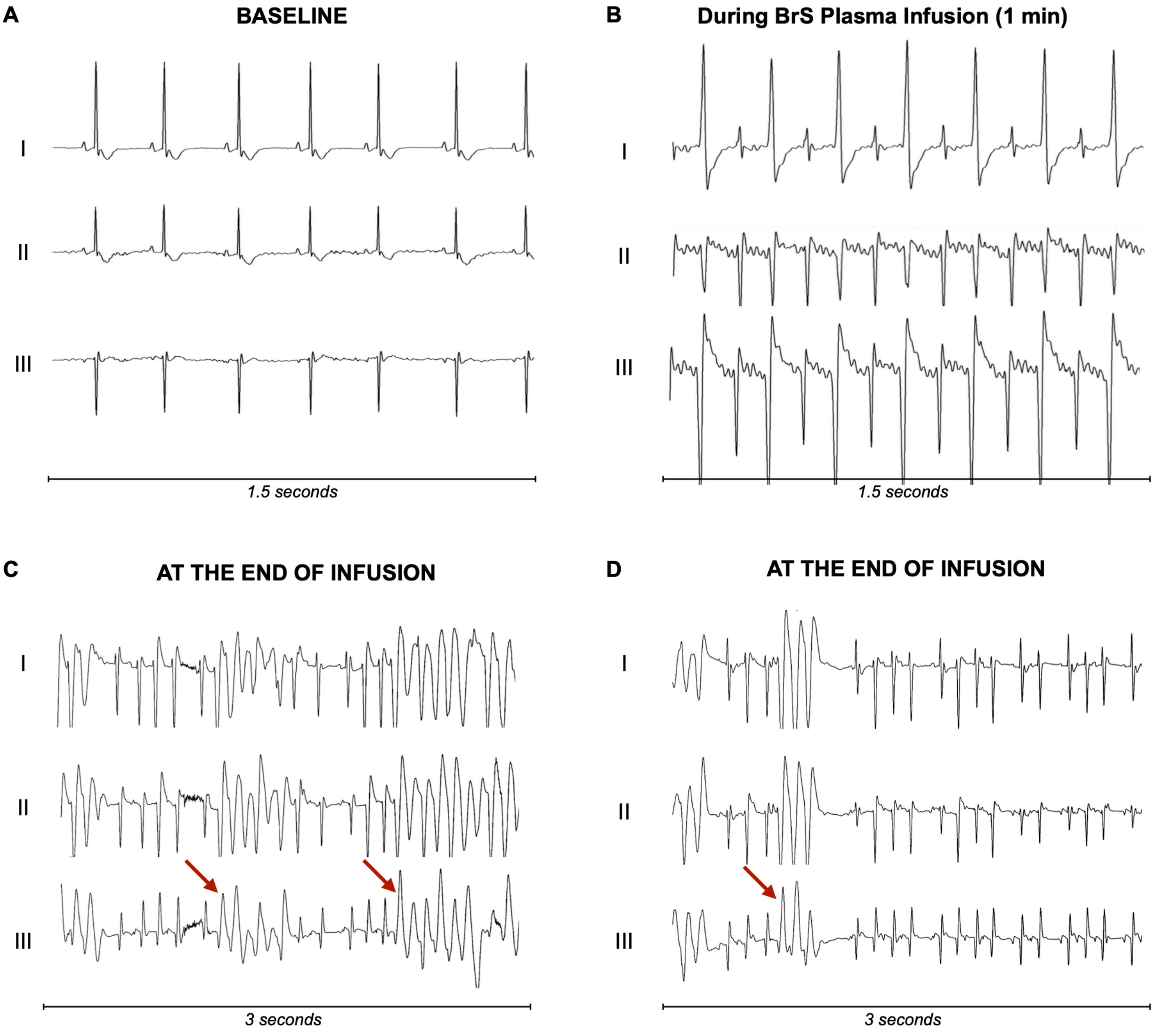
A
BASELINE
I
II
III
1.5 seconds
B
During BrS Plasma Infusion (1 min)
I
II
III
1.5 seconds
C
AT THE END OF INFUSION
I
II
III
3 seconds
D
AT THE END OF INFUSION
I
II
III
3 seconds

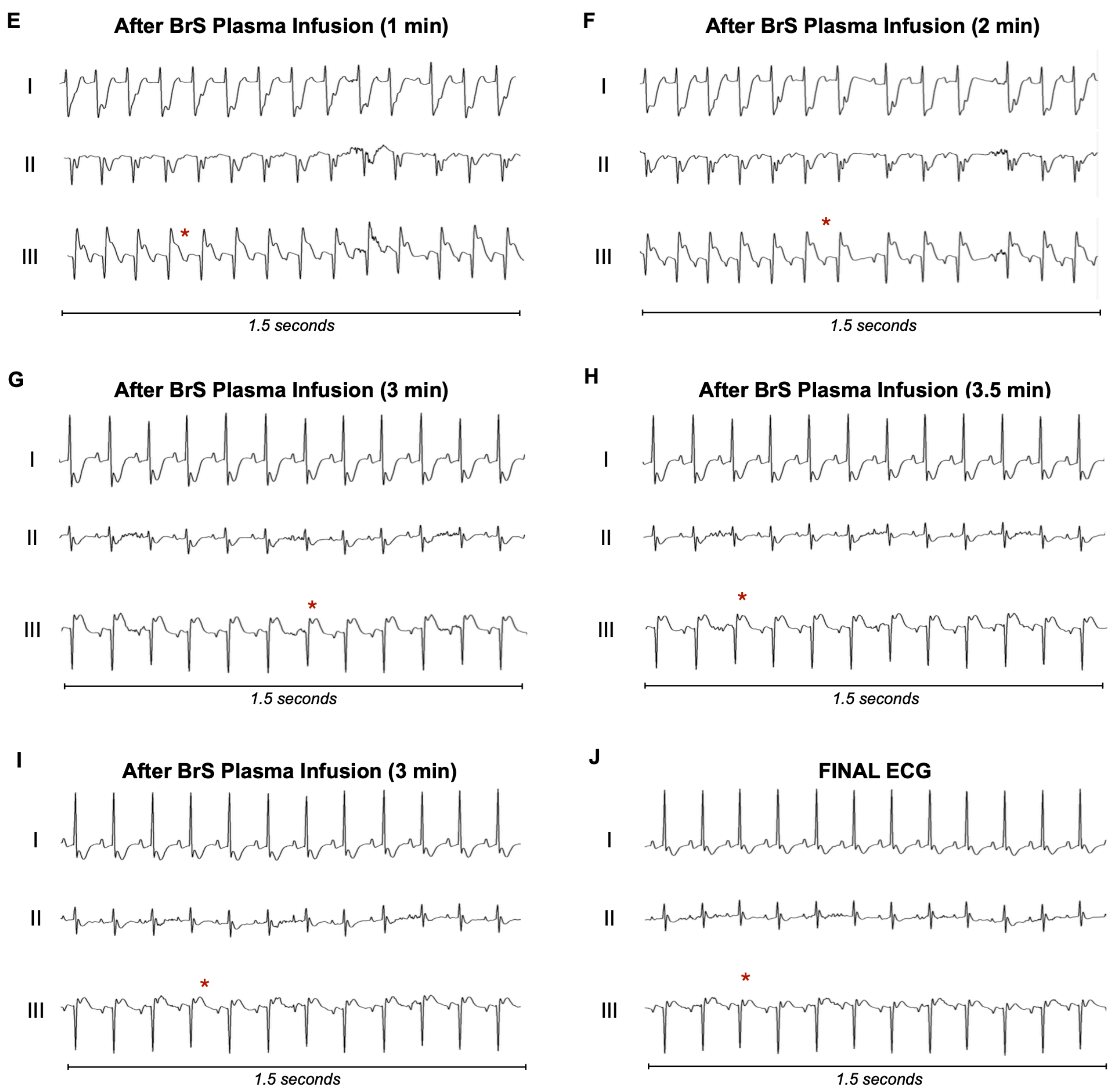

Supplementary Figure 14. Experimental mouse #3 exposed to BrS patient plasma. Sequential electrocardiographic changes in a mouse model following intravenous infusion of plasma from a BrS patient. The panels demonstrate a continuum of arrhythmic manifestations culminating in severe ventricular arrhythmias and coved-type Brugada-like pattern following exposure to BrS patient plasma. The ECG recordings are shown in a 1.5-second time span **(panels A-B, F-J)** and 3-second time span **(panels C-D),** respectively. (**A**) Baseline ECG with normal sinus rhythm; (**B**) Frequent premature ventricular complexes (asterisk) with ventricular bigeminy during plasma infusion, indicating a proarrhythmic effect, and ST-segment elevation (red arrow); (**C** and **D**) Few minutes after infusion, the ECG shows non-sustained torsade-de-points; (**E** and **F**) After 5 minutes, prominent coved-type ST-segment elevation emerges with specular ST depression; (**G-J**) progressive resolution of the ST-segment abnormalities. Same ECG lead configuration as in Figure 4 of the main text.

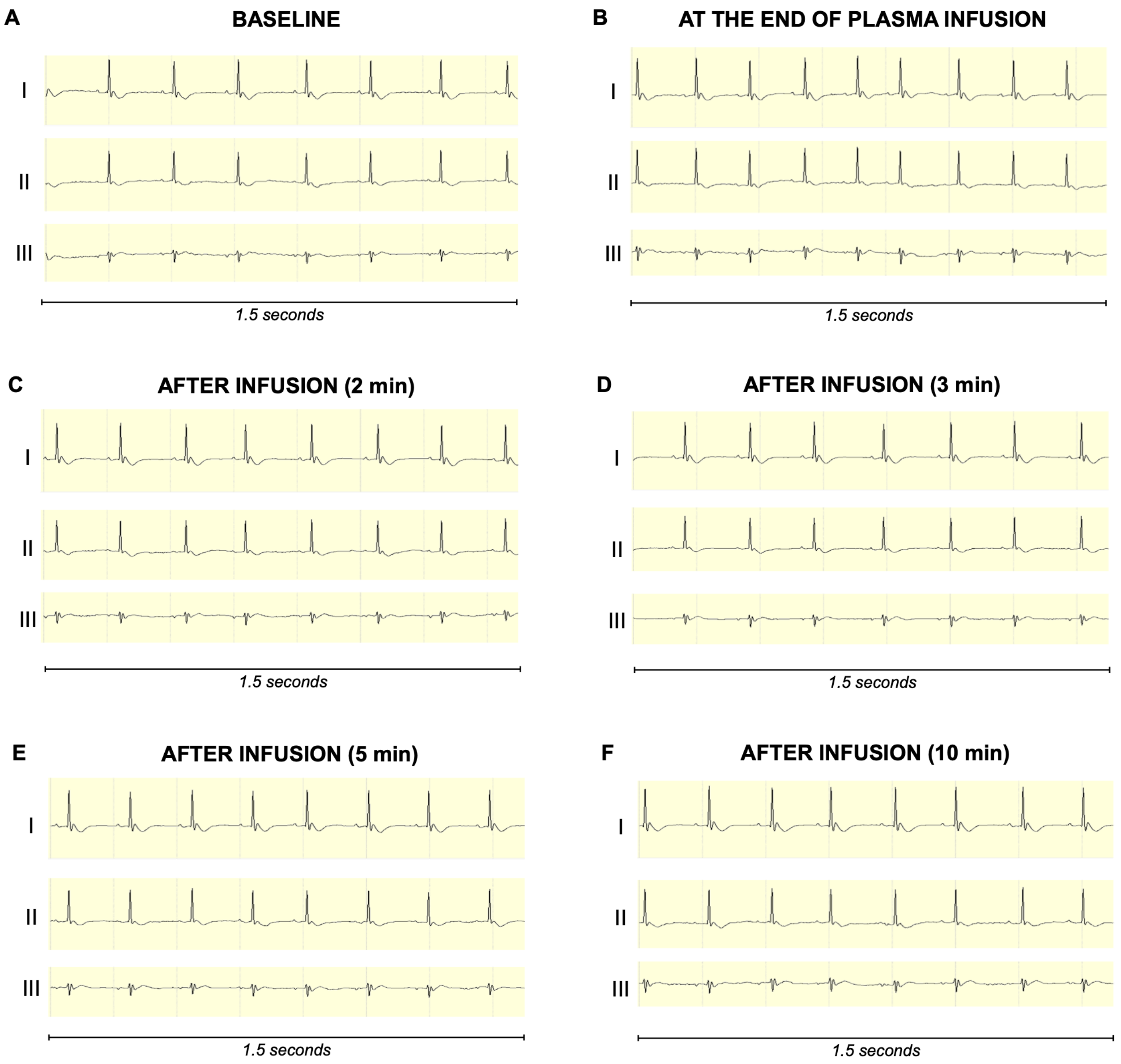

Supplementary Figure 15. Experimental mouse #4 exposed to Control plasma. Experimental control mouse #4 exposed to a control subject plasma. The ECG recordings are shown in a 1.5-second time span. Baseline ECG of control mouse #4, showing normal sinus rhythm and electrical activity prior to (**A**) and at the end of the plasma infusion (**B**). The ECG consistently shows a normal sinus rhythm without any proarrhythmic effects throughout the observation time at two (**C**), three (**D**), five (**E**) and ten (**F**) minutes after the infusion, contrasting with the progressive arrhythmogenic changes seen in mice subjected to the BrS plasma. At the end of the experiment the mouse was still alive. Same ECG lead configuration as in Figure 4 of the main text.

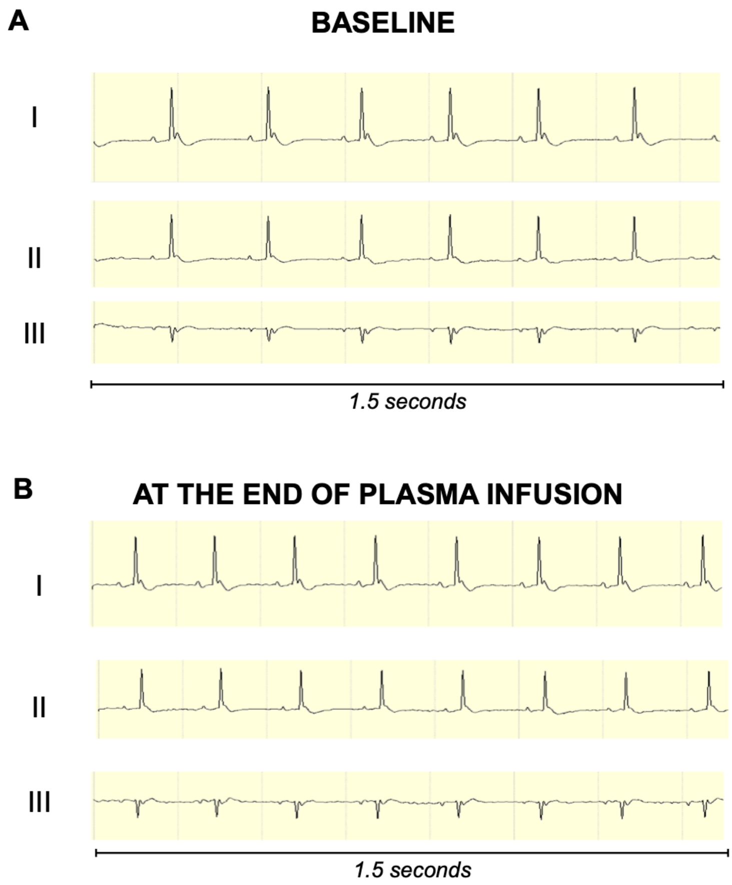

Supplementary Figure 16. Experimental mouse #5 exposed to Control plasma. Experimental control mouse #5 exposed to a control subject plasma (**A**) depicts the baseline ECG of the second control mouse, displaying normal sinus rhythm before plasma infusion; (**B**) shows the ECG after the completion of plasma infusion. No significant ECG changes were observed, indicating the absence of arrhythmogenic effects from the control plasma. The mouse remained alive and stable post-infusion, underscoring the non-arrhythmogenic nature of the control plasma. Same ECG lead configuration as in Figure 4 of the main text.

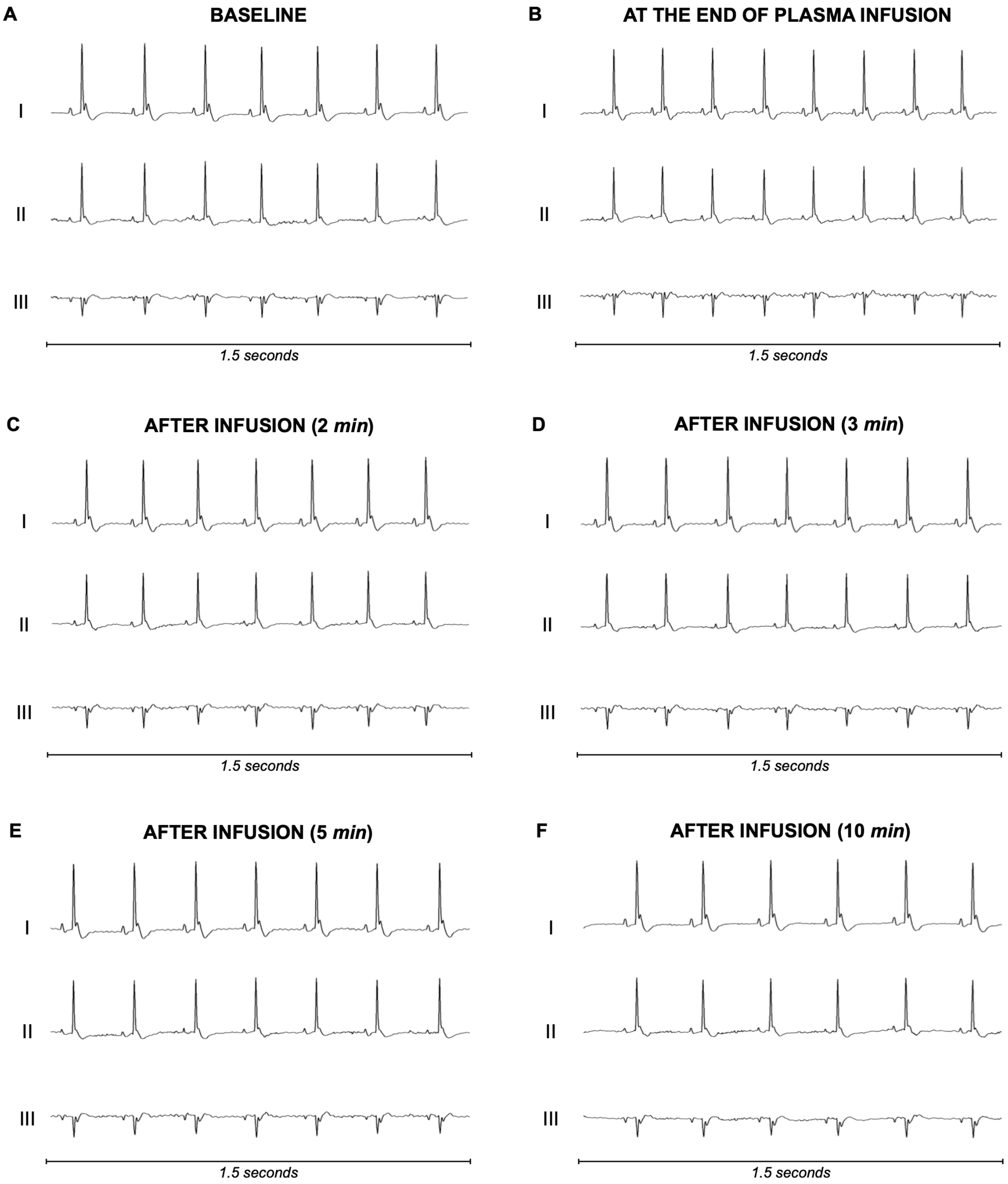

Supplementary Figure 17. Experimental mouse #6 exposed to Control plasma.

Experimental control mouse #6 exposed to a control subject plasma. The ECG recordings are shown in a 1.5-second time span. Baseline ECG shows normal sinus rhythm prior to (**A**) and at the end of the plasma infusion (**B**). No ECG changes occurred overtime. The panels c-f show a normal sinus rhythm without any proarrhythmic effects throughout the observation time at two (**C**), three (**D**), five (**E**) and ten (**F**) minutes after the infusion. At the end of the experiment the mouse was still alive. Same ECG lead configuration as in Figure 4 of the main text.

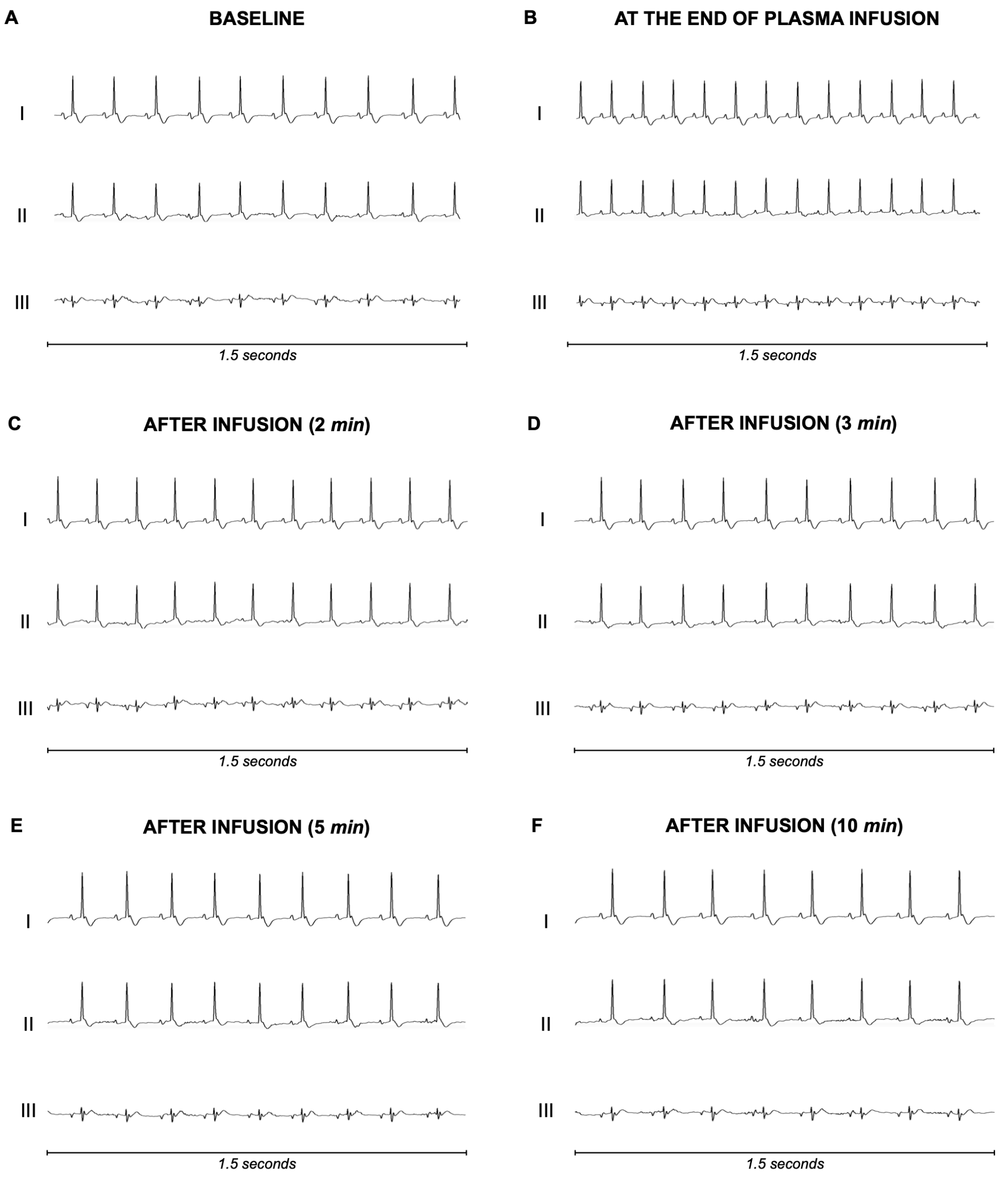

Supplementary Figure 18. Experimental mouse #7 exposed to Antibodies-depleted BrS plasma. This figure shows ECG recording in experimental mouse #7 exposed to plasma from a BrS patient after antibody depletion. The plasma is from the same BrS patient whose plasma, when inoculated in mouse #3, led to the expression of a BrS phenotype. The ECG recordings are shown in a 1.5-second time span. Baseline ECG of this mouse shows normal sinus rhythm and electrical activity prior to (**A**) and at the end of the plasma infusion (**B**). The ECG consistently shows a normal sinus rhythm without any proarrhythmic effects throughout the observation time at two (**C**), three (**D**), five (**E**) and ten (**F**) minutes after the infusion, contrasting with the progressive arrhythmogenic changes seen in mice subjected to the BrS plasma. At the end of the experiment the mouse was still alive. Same ECG lead configuration as in Figure 4 of the main text.

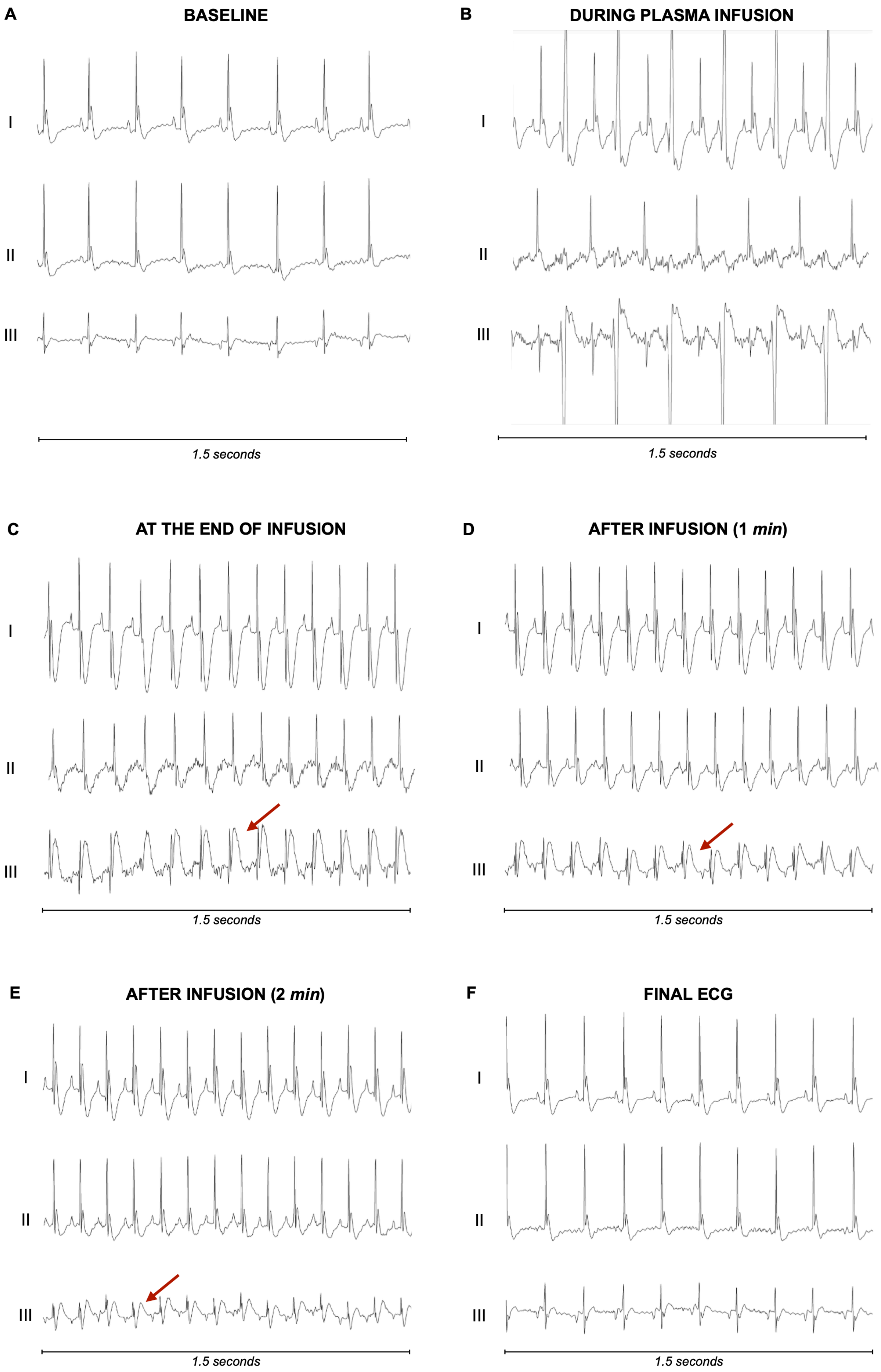
A
BASELINE
B
DURING PLASMA INFUSION
I
II
III
1.5 seconds
C
AT THE END OF INFUSION
D
AFTER INFUSION (1 *min*)
E
AFTER INFUSION (2 *min*)
F
FINAL ECG

Supplementary Figure 19. Experimental mouse #8 exposed to BrS patient plasma. Sequential electrocardiographic changes in a mouse model following intravenous infusion of plasma from a BrS patient. The panels demonstrate a coved-type Brugada-like pattern following exposure to BrS patient plasma (**A-F**). At the end of the infusion prominent coved-type ST-segment elevation emerges with specular ST depression (**C-D**); (**E-F**) progressive resolution of the ST-segment abnormalities. Same ECG lead configuration as in Figure 4 of the main text.

Supplementary Table 1. Additional clinical characteristics and echocardiographic features of the study population.

	BrS (n=50)	Controls (n=50)	p-value
**Comorbidities**			
**Arterial Hypertension, n (%)**	6 (12)	7 (14)	1.000
**Coronary Artery Disease, n (%)**	0 (0)	2 (4)	0.495
**Non malignant arrhythmias , n (%)**	2 (4)	3 (6)	1.000
Atrial Fibrillation, n (%)	0 (0)	1 (2)	1.000
Supraventricular Arrhythmias, n (%)	1 (2)	1 (2)	1.000
Premature Ventricular Complexes, n (%)	1 (2)	1 (2)	1.000
**Diabetes, n (%)**	1 (2)	3 (6)	0.617
**Dyslipidemia, n (%)**	3 (6)	5 (10)	0.715
**Obesity, n (%)**	1 (2)	3 (6)	0.617
**Thyroid Disease, n (%)**	1 (2)	4 (8)	0.362
**Asthma/COPD, n (%)**	0 (0)	1 (2)	1.000
**GERD, n (%)**	2 (4)	1 (2)	1.000
**Echocardiographic parameters**			
**Left ventricular function, mean±SD**	60.2±1.8	59.8±1.7	0.256
**Left atrial dimension, mean±SD**	22.8±2.6	23.3±2.1	0.293
**Diastolic dysfunction, n (%)**	2 (4)	4 (8)	0.677
**Mitral regurgitation (< moderate), n (%)**	1 (2)	3 (6)	0.617
**Mitral regurgitation (> moderate), n (%)**	0 (0)	1 (2)	1.000
**Pharmacological Therapies**			
**Antiplatelet agents, n (%)**	3 (6)	5 (10)	0.715
**Beta blockers, n (%)**	3 (6)	6 (12)	0.487
**Antiarrhythmic drugs, n (%)**	1 (2)	2 (4)	1.000
**ACE inhibitors, n (%)**	3 (6)	3 (6)	1.000
**Sartans, n (%)**	2 (4)	3 (6)	1.000
**Diuretics, n (%)**	1 (2)	2 (4)	1.000
**PPI, n (%)**	3 (6)	3 (6)	1.000
**Statins, n (%)**	3 (6)	5 (10)	0.715

Abbreviations: COPD Chronic obstructive pulmonary disease, GERD gastroesophageal reflux disease, , SD Standard Deviation

Supplementary Table 2. Clinical Characteristics of BrS patients submitted for the epitope mapping.

	**BrS (n=20)**
**Male, n (%)**	13 (65)
**Age (years), mean +/- SD**	40.8±11.2
**Spontaneous type 1 ECG pattern, n (%)**	6 (30)
**Family history of Sudden Death, n (%)**	9 (45)
**Family history of BrS, n (%)**	8 (40)
**Cardiac Arrest or VT/VF, n (%)**	3 (15)
**Arrhythmic Syncope, n (%)**	7 (35)
***SCN5A* variant, n (%)**	2 (10)

SD, standard deviation; VT/VF, ventricular tachycardia/fibrillation.

Supplementary Table 3. Characteristics and Clinical Profiles of Control Cohort Plasma from Non-Brugada Syndrome Cardiovascular Conditions.

ID	Diagnosis	Gender	Age*	LVEF§	Etiology	Genotype	Family history of SD	Other Comorbidities	Drug therapy
1	DCM	M	43	30%	idiopathic	TTN (c.43747+1G>T)	Yes	none	Beta blockers, ARNI, Diuretics, NOAC
2	DCM	F	53	30%	idiopathic	DSP (c.7248del)	no	Hypertension, atrial fibrillation	NOAC, diuretics, Sartans
3	DCM	M	41	35%	idiopathic	TTN (c.72777_72783 del)	no	Atrial fibrillation	NOAC, beta-blocker, Sartans, statin
4	DCM	F	45	35%	idiopathic	FLNC (c.2860_2861del insCT)	no	no	Beta blockers, ARNI, Diuretics
5	DCM	M	60	38%	idiopathic	NA	no	Atrial fibrillation	Beta blockers, ARNI, SGLT2i, Statin, NOAC
6	DCM	F	50	40%	idiopathic	NA	yes	no	Amiodarone, ARNI, diuertics
7	DCM	M	61	30%	CAD	NA	no	no	Beta blockers, ARNI, SGLT2i, Statin, DAPT, diuretics
8	DCM myocarditis	M	35	40%	myocarditis	NA	no	Atrial fibrillation	ACE-i
9	DCM	M	57	20%	CAD	NA	no	no	Asprin, beta-blocker, ARNI, diuretics
10	DCM	F	78	30%	CAD	NA	no	COPD, CKD	VKA, beta-blocker, sartan, diuretics
11	DCM	M	71	25%	CAD	NA	no	Atrial fibrillation	Amiodarone, NOAC, beta-blocker, copidogrel, diuretics, statin, sartan
12	DCM	F	73	30%	CAD	NA	yes	no	Amiodarone, beta-blocker, ARNI, SGLT2i, Statin, diuretics, NOAC
13	DCM	M	68	35%	CAD	NA	no	dislipidemia	Beta blockers, DAPT, ACE-I, SGLT2i, Statin
14	DCM	M	79	35%	CAD	NA	no	Hypertension, diabetes, dislipidemia	Beta blockers, aspirin, ACE-I, SGLT2i, metformin, Statin
15	DCM	M	56	35%	CAD	NA	no	Hypertension, diabetes, dislipidemia	Beta blockers, aspirin, ACE-I, SGLT2i, metformin, Statin
16	DCM	M	47	30%	CAD	NA	no	Hypertension, diabetes, dislipidemia, atrial fibrillation	Beta blockers, ARNI, Diuretics, SGLT2i, statin, NOAC

17	DCM	M	75	30%	CAD	NA	no	Hypertension, dislipidemia, atrial fibrillation, PVCs	Beta blockers, amiodarone, aspirin, diuretics, ACE-I, statin
18	DCM	M	70	30%	CAD	NA	no	Atrial fibrillation, Dislipidemia, Hypertension,	amiodarone, aspirin, ca-antagonist, diuretics, ACE-I, statin
19	DCM	M	64	30%	CAD	NA	no	Atrial fibrillation,	Beta-blocker, NOAC, Statin, ACE-I
20	HCM	M	41	>60%	Hypertrophic CM	MYBPC3 (c.1790G>A)	no	NA	no
21	HCM	M	54	>60%	Hypertrophic CM	MYH7 (c.2631G>T)	no	Atrial fibrillation	Beta-blocker, NOAC
22	HCM	M	62	>55%	Hypertrophic CM	PKP2 (c.1881del)	no	Atrial fibrillation<br><br>Hypertension	NOAC, ACE-I, Beta-blocker
22	DCM	M	72	30%	CAD	NA	no	Hypertension Dyslipidemia	DAPT, statin, ACE-I, Diuretics, SGLT2-i
23	DCM	M	76	40%	CAD	NA	no	Atrial fibrillation	Beta-blocker, NOAC, ACE-I,Diuretics, SGLT2-i
24	DCM	M	68	35%	Tachycardia-induced CM	NA	no	Atrial fibrillation, Hypertension, dyslipidemia, OSAS	Beta blockers, ARNI, SGLT2i, Statin, NOAC,diuretics
25	DCM	M	58	35%	Tachycardia-induced CM	NA	no	Atrial fibrillation	Beta blockers, ARNI, SGLT2i, NOAC, diuretics
26	DCM	M	47	40%	Tachycardia-induced CM	NA	no	Atrial fibrillation	Beta blockers, ARNI, SGLT2i, NOAC, diuretics
27	LQTS	M	43	>55%	Channelopathy	NA	yes	no	Beta-blocker
28	LQTS	M	21	>55%	Channelopathy	KCNQ1 (c.905C>T)	yes	no	Beta-blocker
29	LQTS	F	41	>55%	Channelopathy	NA	yes	no	Beta-blocker
30	LQTS	F	51	>55%	Channelopathy	NA	yes	no	Beta-blocker
31	LQTS	F	36	>55%	Channelopathy	KCNQ1 (c.1760C>T)	yes	no	Beta-blocker
32	LQTS	F	46	>55%	Channelopathy	KCNQ1 (c.794C>T)	yes	GERD	Beta-blocker
33	LQTS	M	46	>55%	Channelopathy	KCNH2 (c.87dupC)	yes	no	Beta-blocker
34	LQTS	F	20	>55%	Channelopathy	KCNQ1 (c.532G>A)	no	no	Beta-blocker
35	LQTS	F	59	>55%	Channelopathy	NA	no	no	Beta-blocker

* Age at time of blood draw; § LVEF at time of diagnosis

Abbreviations: CAD Coronary Artery Disease; CM Cardiomyopathy; DCM Dilated Cardiomyopathy; HCM Hypertrophic Cardiomyopathy; LQTS Long-QT Syndrome

Supplemental Table 4. Clinical Characteristics of BrS patients with and without autoantibodies vs NaV1.5.

	IgG +ve (n=45)	IgG -ve (n=5)	p-value
**Male, n (%)**	30 (66%)	2 (40%)	0.33
**Age (years), mean +/- SD**	39.7±13.5	39.6±12.4	0.98
**Spontaneous type 1 ECG pattern, n (%)**	15 (33%)	0	0.31
**Family history of Sudden Death, n (%)**	23 (51%)	3 (60%)	1.00
**Family history of BrS, n (%)**	19 (42%)	3 (60%)	0.64
**Cardiac Arrest or VT/VF, n (%)**	9 (20%)	0	0.57
**Arrhythmic Syncope, n (%)**	15 (33%)	1 (20%)	1.00
***SCN5A* variant, n (%)**	7 (15%)	0	1.00

Abbreviations: SD, standard deviation; VT/VF, ventricular tachycardia/fibrillation.

Supplementary Table 5. IgG-dependent effect of BrS patient plasma on NaV1.5 current.

	Untreated	BrS plasma w IgG	BrS plasma w/o IgG
n (cells), N (plasma)	n= 24	n=25, N=3	n=36, N=3
Peak Density @ -20 mV (pA/pF)	-249.2 ± 19.7	-181.1 ± 16.8*	-281.6 ± 19.6*
Activation curve $V_{1/2}$ (mV)	-32.3± 0.8	-31.7±1.0	-35.6±0.7*
Activation curve k (mV)	6.9±0.2	7.3±0.3	6.4±0.2
Inactivation curve $V_{1/2}$ (mV)	-80.2±1.7	-80.2±1.4	-82.6±1.1
Inactivation curve k (mV)	-6.0± 0.1	-6.0±0.1	-5.9±0.1

*=p<0.5

Supplementary Table 6 Values of current density, $V_{1/2}$ and k for TTX-sensitive and Nifedipine-sensitive currents in hiPS-CM untreated or incubated with BrS plasma.

	Sodium current (TTX-sensitive)	
	Untreated	BrS Plasma
n (cells) N (plasma)	n= 26	n=33, N=3
Peak Density @ -10 mV (pA/pF)	-95.5±10.8	-48.3±6.4*
Activation curve $V_{1/2}$ (mV)	-24.4±0.9	-22.2±0.8
Activation curve k (mV)	6.4±0.2	6.7±0.2
	Calcium current (Nifedipine-sensitive)	
	Untreated	BrS Plasma
n (cells) N (plasma)	n= 22	n=30, N=3
Peak Density @ 0 mV	-22.0±1.6	-22.9±1.4
Activation curve $V_{1/2}$ (mV)	-15.7±1.0	-16.3±1.3
Activation curve k (mV)	7.8±0.3	7.6±0.3

*=p<0.5

Supplementary Table 7. Values of current density, $V_{1/2}$ and k for NaV1.5 or Cav3.2 T-type calcium current when the channels were overexpressed in HEK293A cells.

	NaV1.5	
	Untreated	BrS Plasma
n (cells), N (plasma)	n= 32, N=3	n=29, N=3
Peak Current Density @ -20 mV (pA/pF)	-327.7 ± 26.4	-251.9 ± 22.0 *
Activation curve $V_{1/2}$ (mV)	-35.7 ± 0.6 mV	-37.1 ± 0.7 mV
Activation curve k (mV)	6.8 ± 0.2	6.8 ± 0.3
Inactivation curve $V_{1/2}$ (mV)	-83.3 ± 1.1 mV	-82.9 ± 1.2 mV
Inactivation curve k (mV)	-6.1 ± 0.1	-5.9 ± 0.7
	Cav3.2	
	Untreated	BrS Plasma
n (cells), N (plasma)	n= 27, N=3	n=32, N=3
Peak Current Density @ -20 mV (pA/pF)	-100.3 ± 7.1	-107.2 ± 8.6
Activation curve $V_{1/2}$ (mV)	-40.0 ± 1.0	-37.3 ± 0.7*
Activation curve k (mV)	7.1 ± 0.2	6.8 ± 0.2
Inactivation curve $V_{1/2}$ (mV)	-90 ± 1.3	-89.5 ± 1.1
Inactivation curve k (mV)	-9.2 ± 0.4	-9.3 ± 0.3

*=p<0.5

Supplementary Table 8. Values of current density, $V_{1/2}$ and k obtained from NaV1.5 and NaV1.4 currents in HEK293A cells.

	NaV1.5	
	Untreated	BrS plasma
n (cells), N (plasma)	n= 15	n=18, N=3
Peak Density @ -20 mV (pA/pF)	-292.1±41.8	-166.0±33.7*
Activation curve $V_{1/2}$ (mV)	-35.5±1.53	-29.9±1.14*
Activation curve k (mV)	6.74±0.32	7.83±0.47
Inactivation curve $V_{1/2}$ (mV)	-72.7±1.33	-73.3±1.28
Inactivation curve k (mV)	-6.77±0.29	-6.98±0.25
	NaV1.4	
	Untreated	BrS Plasma
n (cells), N (plasma)	n= 23	n=31, N=3
Peak Density @ 0 mV (pA/pF)	-126.1±12.7	-53.6±6.1
Activation curve $V_{1/2}$ (mV)	-18.6±0.9	-13.9±0.7*
Activation curve k (mV)	7.89±0.21	8.25±0.27
Inactivation curve $V_{1/2}$ (mV)	-63.0±0.4	-62.7±0.67
Inactivation curve k (mV)	-6.20±0.20	-7.15±0.29

*=p<0.5

References

1. Zeppenfeld K, Tfelt-Hansen J, de Riva M, Winkel BG, Behr ER, Blom NA*, et al.* 2022 ESC Guidelines for the management of patients with ventricular arrhythmias and the prevention of sudden cardiac death. *Eur Heart J* 2022;**43**:3997-4126. doi: 10.1093/eurheartj/ehac262
2. Antzelevitch C, Brugada P, Borggrefe M, Brugada J, Brugada R, Corrado D*, et al.* Brugada syndrome: report of the second consensus conference: endorsed by the Heart Rhythm Society and the European Heart Rhythm Association. *Circulation* 2005;**111**:659-670. doi: 10.1161/01.CIR.0000152479.54298.51
3. Calamaio S, Serzanti M, Boniotti J, Fra A, Garrafa E, Cominelli M*, et al.* Human iPSC-Derived 3D Hepatic Organoids in a Miniaturized Dynamic Culture System. *Biomedicines* 2023;**11**. doi: 10.3390/biomedicines11082114
4. Uzun AU, Mannhardt I, Breckwoldt K, Horvath A, Johannsen SS, Hansen A*, et al.* Ca(2+)-Currents in Human Induced Pluripotent Stem Cell-Derived Cardiomyocytes Effects of Two Different Culture Conditions. *Front Pharmacol* 2016;**7**:300. doi: 10.3389/fphar.2016.00300
5. Liao Y, Wang J, Jaehnig EJ, Shi Z, Zhang B. WebGestalt 2019: gene set analysis toolkit with revamped UIs and APIs. *Nucleic Acids Res* 2019;**47**:W199-W205. doi: 10.1093/nar/gkz401
6. Kim H, de Jesus AA, Brooks SR, Liu Y, Huang Y, VanTries R*, et al.* Development of a Validated Interferon Score Using NanoString Technology. *J Interferon Cytokine Res* 2018;**38**:171-185. doi: 10.1089/jir.2017.0127
7. Marksteiner J, Ebner J, Salzer I, Lilliu E, Hackl B, Todt H*, et al.* Evidence for a Physiological Role of T-Type Ca Channels in Ventricular Cardiomyocytes of Adult Mice. *Membranes (Basel)* 2022;**12**. doi: 10.3390/membranes12060566
